# Absence of HIV-1 Evolution in the Gut-Associated Lymphoid Tissue from Patients on Combination Antiviral Therapy Initiated during Primary Infection

**DOI:** 10.1371/journal.ppat.1002506

**Published:** 2012-02-02

**Authors:** Teresa H. Evering, Saurabh Mehandru, Paul Racz, Klara Tenner-Racz, Michael A. Poles, Amir Figueroa, Hiroshi Mohri, Martin Markowitz

**Affiliations:** 1 Aaron Diamond AIDS Research Center, an affiliate of the Rockefeller University, New York, New York, United States of America; 2 Bernhard-Nocht Institut Fur Tropenmedizin, Hamburg, Germany; 3 Departments of Medicine, Microbiology and Pathology, New York University Medical Center, New York, New York, United States of America; King's College London School of Medicine, United Kingdom

## Abstract

Mucosal mononuclear (MMC) CCR5+CD4+ T cells of the gastrointestinal (GI) tract are selectively infected and depleted during acute HIV-1 infection. Despite early initiation of combination antiretroviral therapy (cART), gut-associated lymphoid tissue (GALT) CD4+ T cell depletion and activation persist in the majority of HIV-1 positive individuals studied. This may result from ongoing HIV-1 replication and T-cell activation despite effective cART. We hypothesized that ongoing viral replication in the GI tract during cART would result in measurable viral evolution, with divergent populations emerging over time. Subjects treated during early HIV-1 infection underwent phlebotomy and flexible sigmoidoscopy with biopsies prior to and 15–24 months post initiation of cART. At the 2^nd^ biopsy, three GALT phenotypes were noted, characterized by high, intermediate and low levels of immune activation. A representative case from each phenotype was analyzed. Each subject had plasma HIV-1 RNA levels <50 copies/ml at 2^nd^ GI biopsy and CD4+ T cell reconstitution in the peripheral blood. Single genome amplification of full-length HIV-1 envelope was performed for each subject pre- and post-initiation of cART in GALT and PBMC. A total of 280 confirmed single genome sequences (SGS) were analyzed for experimental cases. For each subject, maximum likelihood phylogenetic trees derived from molecular sequence data showed no evidence of evolved forms in the GALT over the study period. During treatment, HIV-1 envelope diversity in GALT-derived SGS did not increase and post-treatment GALT-derived SGS showed no substantial genetic divergence from pre-treatment sequences within transmitted groups. Similar results were obtained from PBMC-derived SGS. Our results reveal that initiation of cART during acute/early HIV-1 infection can result in the interruption of measurable viral evolution in the GALT, suggesting the absence of *de-novo* rounds of HIV-1 replication in this compartment during suppressive cART.

## Author Summary

This study was undertaken to determine if the gastrointestinal tract is a site of ongoing viral replication during suppressive combination antiretroviral therapy (cART) (defined by plasma HIV-1 RNA levels below 50 copies/ml). We found no evidence of substantial viral evolution in HIV-1 envelope sequences derived from peripheral blood mononuclear cells or cells of the gastrointestinal tract lymphoid tissue in participants initiating cART during early HIV-1 infection. To our knowledge, this is the first application of the single genome amplification technique to the comparative analysis of HIV-1 quasi-species derived from the gastrointestinal tract, demonstrating that in these individuals, cART has the ability to halt measurable evolution of HIV-1 envelope in this compartment. These findings suggest the absence of *de-novo* rounds of HIV-1 replication during suppressive cART and by extension, that experimentally observed, persistently elevated levels of immune activation in the gastrointestinal lymphoid tissue seen after the early initiation and uninterrupted use of cART (despite relative immune reconstitution in the blood) is likely due to factors other than ongoing viral replication. This implies that in this virally suppressed population, cART intensification is unlikely to significantly impact persistent CD4+ T cell depletion or increased levels of immune activation in the gastrointestinal tract.

## Introduction

Acute infection with human immunodeficiency virus type 1 (HIV-1) is a critical time during which host factors including innate and adaptive immunity converge with virologic characteristics to determine the course of clinical progression in infected individuals [1]–[3]. In the absence of combination antiretroviral therapy (cART), HIV-1 infection is maintained in a chronic state- characterized by high levels of viral production with associated immune activation that is responsible in large part for progressive CD4+ T cell depletion [4]. Repeated rounds of infection of susceptible CD4+ cells of various types occur with continued generation of long-lived cells harboring replication competent virus, and that persist during therapy [5].

The HIV-1 treatment landscape was transformed when in 1996, highly active antiretroviral therapy (HAART) became the standard of care for the treatment of HIV-1 infection. The advent of HAART is directly credited with the retardation of the overall progression of HIV infection to AIDS as well as the progression of AIDS to death. The end result has been a considerable decrease in morbidity, and increase in survival after AIDS diagnosis [6], [7]. Combination antiretroviral drug therapy (cART) can dramatically suppress HIV replication and reduce the plasma HIV-1 viral load in compliant patients, resulting in immune reconstitution of memory CD4+ and CD8+ T cells and the restoration of T cell immunity [8]–[11]. Despite these advances, current regimens remain unable to eliminate the reservoir of latent virus in resting CD4+ T lymphocytes. As a result, cessation of therapy predictably results in the resurgence of virus replication [12]–[17].

The gut-associated lymphoid tissue (GALT) contains the vast majority, and most complex pool of immune cells [18]. In addition, intrinsic characteristics of the mucosal compartment, including the predominance of activated and well differentiated gastrointestinal (GI) mucosal CD4+ T cells with a memory phenotype [19], [20] permit HIV-1 infection and accommodate its replication.

Several studies have examined the critical role played by the GI tract in early simian immunodeficiency (SIV) pathogenesis. In the SIV/macaque model, SIV challenge has been shown to result in early, profound depletion of GI mucosal CD4+ lymphocytes [21]–[23]. Additional studies in the SIV/macaque model revealed that up to 60% of memory CD4+CCR5+ T cells may be selectively infected and lost during this early stage of infection [24].

We and others have shown that the human GI lymphoid system is similarly targeted during acute and early HIV-1 infection. A greater percentage of mucosal CD4+ lymphocytes express the CCR5 chemokine coreceptor [25]–[28] when compared to the peripheral blood. Preferential targeting of the CCR5+ memory CD4+ T cell subset therefore results in substantial depletion of CD4+ T cells in the gut-associated lymphoid tissue (GALT). As a consequence, up to 60% of CD4+ T cells in the lamina propria of the lower gastrointestinal (GI) tract are lost as early as 2–4 wk after infection [29]–[31]. Cross-sectional analysis of a cohort of primary HIV-1 infection subjects demonstrated that despite immune reconstitution in the peripheral blood mononuclear cells (PBMCs), persistent CD4+ T cell depletion and immune activation have been noted in the GALT in a majority of patients despite up to 5 years of suppressive cART [32], [33].

The latent reservoir of HIV-1 is established early in infection and persists despite the initiation of cART during the primary phase of infection [12], [13], [34]. Employing a real-time reverse transcriptase-initiated PCR single copy assay (SCA), Palmer et al. have demonstrated that the majority of infected individuals on “suppressive” ART (defined by plasma HIV-1 RNA levels <50 copies/mL) have persistent HIV-1 viremia below the current limit of detection of commercially available, and currently less sensitive assays. This real-time RT-initiated PCR assay quantifies HIV-1 RNA concentration down to 1 copy/mL of plasma with use of an internal control for viral pelleting and extraction followed by separate RT-PCR reactions for HIV-1 and virion control sequences [35].

Numerous studies have attempted to determine if measurable low-level residual viremia during “suppressive” cART is the result of ongoing cycles of HIV-1 replication. Studies have suggested that ongoing viral replication in patients on apparently suppressive cART may occur [36]–[46]. These include reports of decreases in low-level viremia with HAART intensification, persistent expression of unspliced (US) HIV-1 mRNA in PBMC, persistence of HIV-1 episomal cDNA in PBMC and modest evolution of viral envelope sequences over time [36], [37], [39], [40]. Conversely and generally more recently, others have found no evidence of ongoing HIV-1 replication during suppressive cART [47]–[53]. These include studies that have generated phylogenetic data suggesting the absence of viral evolution in patients undergoing successful cART and support the notion that the reservoir is intrinsically stable [51]–[53]. Bailey et al. investigated plasma and cellular viral sequences obtained during suppressive cART and found that in some patients on antiretroviral therapy, the prolonged production of a small number of viral clones without evident evolution is a major mechanism for persistent viremia [47]. Most recently, *in-vitro* work by Sigal et al. proposes the possibility of cell to cell transmission as a mechanism of ongoing HIV-1 replication despite ART [54]. As a result, the important question of whether or not there is ongoing HIV-1 replication despite “suppressive” cART remains under debate.

We hypothesized that one cause of persistent immune activation and CD4+ T cell depletion in the gastrointestinal lymphoid compartment despite immune reconstitution in the blood may be ongoing local HIV-1 replication in the GALT during apparently suppressive cART, which could potentially constitute a source for replenishment of the latently infected resting CD4+ T cell pool.

We therefore endeavored to measure qualitatively and quantitatively the degree of HIV-1 evolution in the PBMC and GALT during cART to determine whether the lymphoid tissue of the GI tract is a reservoir of ongoing viral replication during suppressive cART. We hypothesized that ongoing viral replication in the GALT during suppressive cART would result in measurable viral evolution, with more divergent populations emerging in the GALT than in the PBMC over time. We also hypothesized that if continued HIV-1 replication in the GALT was responsible for the persistent immune activation in this compartment, the amount of HIV-1 *env* evolution would correlate positively with experimentally determined levels of immune activation, while correlating negatively with levels of immune reconstitution in the GALT.

This research utilizes the single genome amplification (SGA) technique to generate HIV-1 variant sequences. This terminal dilution technique uses a single molecule of DNA or cDNA as the template for amplification and sequencing [55]–[58]. SGA has the ability to achieve a degree of detection of diversity greater than 20% within a repeatedly sampled viral population [59] and has been shown to decrease taq-induced recombination, template resampling, nucleotide misincorporation and cloning bias when compared to more conventional sequencing methods [55]–[57], [60]–[62]. The use of this method therefore produces a more accurate representation of in-vivo HIV-1 quasispecies than compared to bulk sequencing methods [63].

The mucosal tissues harbor the primary targets of HIV-1 infection and serve as an important route for HIV-1 entry and replication. For these reasons, understanding the spectrum of HIV-1 quasispecies in the GI mucosa may be critical in revealing determinants of viral entry, persistence and developing treatment strategies to improve viral suppression and overcome an established obstacle to eradicate HIV-1 in the infected host.

## Results

### Immunologic and virologic characteristics of the study groups

In the experimental group, contemporaneous peripheral-blood and recto-sigmoid colonic mucosal tissue samples were obtained from three cART-naïve, HIV-1 seropositive males identified during acute/early HIV-1 infection (Table 1). Combination ART was initiated in this group within 72 hrs of flexible sigmoidoscopy. Experimental subjects reported >95% uninterrupted adherence to cART at the time of repeat phlebotomy and flexible sigmoidoscopy with biopsies 15 to 25 months post initiation of cART (Table 1). Levels of immune activation and immune reconstitution following cART for these individuals had been previously determined at the time of initial sample collection using flow cytometry and immunohistochemistry respectively.

**Table 1:** Clinical profile and ARV treatment history for study participants and positive controls.

Participant	HIA	IIA	LIA	POS1	POS2
**Elisa at Time Point #1 (TP1)**	+	+	+	+	+
**Western Blot TP1**	+	+	+	+	+
**PBMC TP1**	12/9/2004	5/5/2004	11/9/2004	3/8/2007	8/10/2006
**GALT TP1**	12/13/2004	5/6/2004	11/9/2004	N/A	N/A
**Estimated Duration of Infection PBMC TP1**	68 days	30 days	88 days	78 days	54 days
**Fiebig Stage TP1**	VI	V	VI	V	V
**CD4 Count cells/ul TP1**	442	250	489	656	560
**Plasma HIV-1 RNA (VL) copies/ml TP1**	148,000	5,560,000	86,100	46,700	72,300
**cART Start Date**	12/16/2004	5/6/2004	11/16/2004	N/A	N/A
**Initial cART regimen**	TDF/FTC+EFV	TDF/FTC+LPV/r	TDF/FTC+EFV	N/A	N/A
**Time to 1st VL <50 copies/ml**	110 days	113 days	119 days	N/A	N/A
**Time to PBMC TP2**	1.3 y (489 d)	1.1 y (414 d)	1.4 y (506 d)	1.9 y (691 d)	1.8 y (670 d)
**Time to GALT TP2**	1.3 y (489 d)	2.1 y (761 d)	1.3 y (483 d)	N/A	N/A
**CD4 Count cells/µl TP2**	774	629 at PBMC TP2	556	486	684
		903 at GALT TP2			
**Plasma VL copies/ml TP2**	<20	<20 at PBMC TP2	<20	27,300	>100,000
		<50 at GALT TP2			

**Figure 1:**
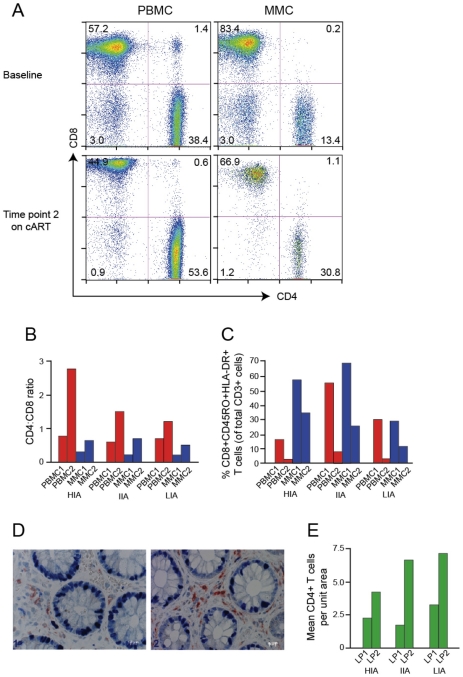
Immunological profiles of experimental patients before and after initiation of cART. (A) Representative flow plots from low immune activator patient (LIA) are depicted. CD8^+^ T cells are shown on the *x*-axis and CD4^+^ T cells on the *y*-axis. (B, C) Bar graphs depicting CD4∶CD8 ratio (B) and levels of immune activation (C) of PBMC (red bars) and MMC (blue bars) before (PBMC1 and MMC1) and after (PBMC2 and MMC2) initiation of antiretroviral therapy. High immune activator (HIA), intermediate immune activator (IIA) and low immune activator (LIA) are compared. (D, E) Immunohistochemical characterization of immune effector sites in rectosigmoid biopsies. Using a PC-based image-analysis system (KS 4000, Kontron) a standard area was set by the image analyzer. A total of between 10 and 15 consecutive non-overlapping fields were analyzed. In figure (D), a biopsy section (viewed at 40× magnification) compares CD4+ T cells (stained red) of subject IIA before (left panel) and after (right panel) initiation of antiretroviral therapy. Figure (E) depicts mean CD4+ T before (LP1) and after (LP2) initiation of antiretroviral therapy. HIA, IIA and LIA are compared.

During early HIV-1 infection, all individuals demonstrated CD4+ T cell depletion at similar levels in the PBMCs, with CD4/CD8 ratios below 1 (Figure 1A, 1B). The CD4/CD8 ratio inversion was more pronounced in the MMC compartment than in the PBMC compartment for all individuals at this early time point. Following 1–2 years of cART, all individuals had successful reconstitution of the peripheral CD4+ T cell compartment, with substantial increases in peripheral CD4+ T cell counts (Table 1) and normalization of the CD4/CD8 ratio. Less successful CD4+ T cell reconstitution in the MMCs was seen, and none of the individuals reconstituted to a CD4/CD8 ratio >1 despite cART (Figure 1B). Immunohistochemistry on GI biopsy specimens further demonstrated the inability of these individuals to fully reconstitute CD4+ T cells of the GI tract through specific examination of the lamina propria (LP), an important immune-effector site where preferential CD4+ T cell depletion occurs during primary HIV infection [30]. Mean CD4+ T cells per unit area were severely depressed during early infection. Following 1–2 years of cART, each individual was able to reconstitute LP CD4+ T cells with varying degrees of success, although none quite to the 11.0+/−3.3 cells/unit area seen by Mehandru et al. in HIV-1 negative individuals [32](Figure 1D, 1E). Similarly, during primary HIV-1 infection prior to initiation of cART, levels of immune activation (as measured by %CD8+CD45RO+HLA-DR+ T cells) were abnormally elevated in the CD8+ PBMCs and MMCs for all subjects (Figure 1C). After >1 year on cART, levels of immune activation in the PBMC compartment dropped dramatically to levels that approximate those seen in HIV-1 negative individuals (4.8%+/−3.9%) [32]. However, in the MMC compartment, while decreased from primary infection, levels of memory CD8+ T cell immune activation remained elevated above that seen in historic HIV-1 negative control individuals (19.8%+/−9.8%) [32], for our High Immune Activator (HIA) at 35.5%. GALT immune activation levels for the Intermediate Immune Activator (IIA) and Low Immune Activator (LIA) were 25.2% and 12.1% respectively (Figure 1C).

Given the importance of the GI LP as a target for CD4+ T cell depletion during primary HIV-1 infection, the link between persistent immune activation in the CD8+ T cell compartment to adverse HIV-1 disease progression [64] and the negative correlation in these three individuals between the ability to reconstitute CD4+ T cells in the GALT and the degree of residual CD8+ T cell activation in the MMCs, subjects were chosen based on the exhibition of one of three phenotypes in the GI lymphoid tissue at the time of 2^nd^ biopsy: (1) High Immune Activation (HIA) with 35.5% CD8+ CD45RO+HLA-DR+ T cells in the mucosal mononuclear cell (MMC) compartment and an absolute CD4+ T cell count of 4.3 cells/unit area in the GI lamina propria (2) Intermediate Immune Activation (IIA) with 25.2% CD8+CD45RO+HLA-DR+ T cells and an absolute CD4+ T cell count of 6.7 cells/unit area and (3) Low Immune Activation (LIA) with 12.1% CD8+CD45RO+HLA-DR+ T cells and an absolute CD4+ T cell count of 7.2 cells/unit area (Figure 1C, 1E). All three had plasma HIV-1 viral loads of <50 copies/ml at the time of the 2^nd^ GI biopsy and CD4+ T cell counts of 774, 903 and 556 cells/ml respectively (Table 1). Repeat testing of stored plasma corresponding to the 2^nd^ PBMC sampling time point revealed all individuals to have plasma HIV-1 viral loads of below 20 copies/ml (Roche Taqman, v. 2.0). For individual IIA, GALT TP2 occurred approximately 1 year after PBMC TP2. Although plasma HIV-1 RNA was determined to be <50 copies/ml at GALT TP2, no PBMCs or plasma were stored at GALT TP2 for retrospective analysis. As a result, although the plasma HIV-1 RNA for IIA one year prior to GALT TP2 was clearly <20 copies/ml, we were unable to determine if the plasma viral load for this individual at GALT TP2 was also <20 copies/ml.

For the control group, peripheral-blood mononuclear cells (PBMCs) were obtained during their presentation with early HIV-1 infection (Table 1). Both participants elected to remain naïve to cART, and were continuously viremic to the second PBMC sampling time point, 1.8 to 1.9 years later. Recto-sigmoid colonic mucosal tissue sampling was not performed for the positive controls.

### Single Genome Amplification and Sequencing of HIV-1 envelope

Single genome amplification (SGA) of full-length HIV-1 *env* (>2.5 kb) was performed on proviral genomic DNA from cryopreserved PBMCs and GALT tissue where indicated for each individual using a modification of the method of Salazar-Gonzalez et al. [63], [65]. A total of 378 confirmed single genome sequences (SGS) from the three experimental and two positive control patients were obtained as described in Methods. On average, 23 SGS were obtained per patient, per time-point, per compartment.

**Figure 2:**
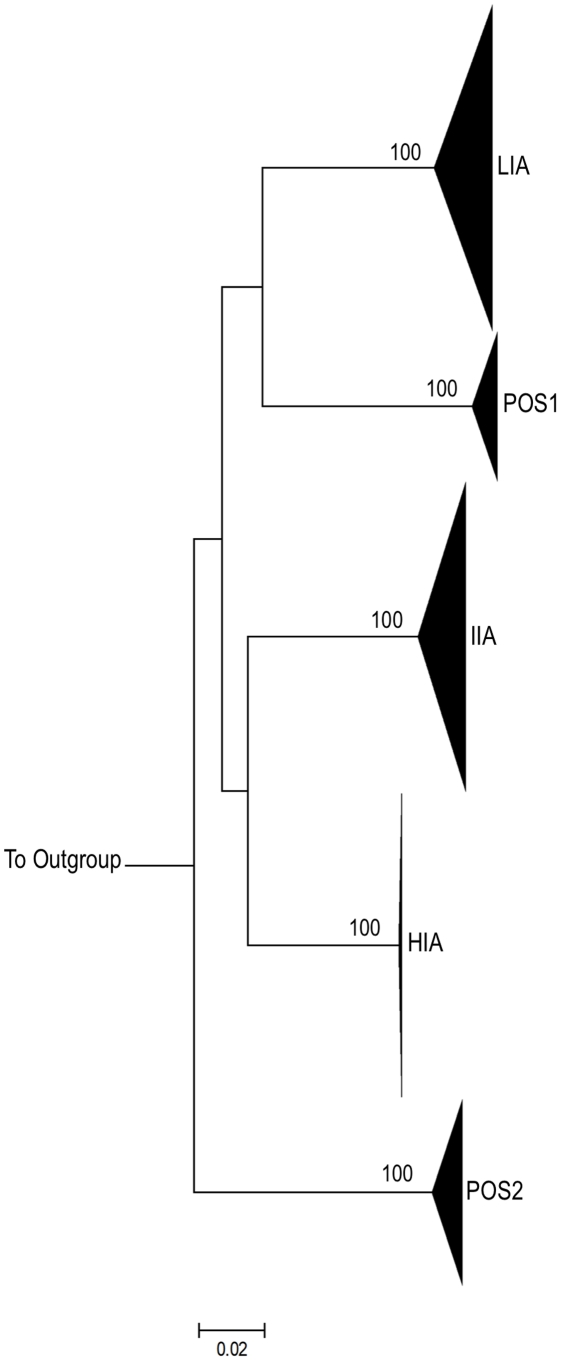
Intra-Patient clustering of HIV-1 *env* quasi-species. Maximum Likelihood (ML) tree depicting full-length HIV-1 *env* sequences from three experimental subjects (HIA, IIA, LIA) and 2 positive control subjects (POS1 and POS2). For each subject, all sequences from both time-points (baseline and on treatment) and from all compartments described are shown. Bootstrap values (>95%) are shown for inter-subject clusters. 1000 bootstrap replicates were run and Bootstrap values over 85% are shown. The horizontal scale bar represents 2.0% genetic distance. Each subject forms a tight cluster and is distinct from other experimental subjects or controls. HXB2 was used as an outgroup.

Phylogenetic analysis demonstrates that sequences from each experimental and control patient form tight and distinct clusters (Figure 2). This is consistent with the absence of contamination between patient samples during PCR [66].

All experimental and control participants were found to harbor HIV-1 Subtype B virus using the REGA HIV-1 subtyping tool [67], [68]. Along with CD4, HIV-1 typically uses the CCR5 chemokine coreceptor for entry early in infection [69]. Genotypic changes allowing the virus to use CXCR4 have been associated with the more rapid progression of HIV-1 disease [70]. Analysis of translated HIV-1 V3 loop sequences for all viral quasispecies from the positive controls and participants HIA and IIA were predicted by the Web Position Specific Scoring Matrix (PSSM) tool (SINSI matrix) to utilize the CCR5 HIV-1 co-receptor for viral entry [71], [72]. Interestingly, translated V3 loop sequences from individual LIA were predicted to use the CXCR4 co-receptor for viral entry. The PSSM bioinformatic method has a reported sensitivity of 84% sensitivity and 96% specificity for the prediction of CXCR4 usage [72]. Given the relative infrequency of transmitted CXCR4 virus [73]–[75], a cyropreserved pre-treatment plasma specimen from LIA was sent for cell-based confirmation of co-receptor usage for entry into CD4+ cells. The LIA plasma sample submitted for the determination of HIV-1 tropism using the monogram assay was collected on the same day as the pre-treatment samples used for SGA of HIV-1 *env* from PBMC and GALT in this individual. Using pseudotyped virus engineered to express the LIA HIV-1 *env*, Trofile assays (Monogram Biosciences) [76] reported a dual/mixed virus population with the ability to use CXCR4 and/or CCR5 co-receptors to enter the CD4+ cell. The reported relative light units (RLUs) for CCR5 and CXCR4 usage in the population were 132,786 and 1,439 respectively (near the limit of detection for CXCR4). Given the uniform predictions for all LIA quasispecies in PSSM scoring, we concluded the LIA viruses were likely to represent a dual mixed virus population with the ability to utilize both co-receptors for cellular entry.

### SGA of HIV-1 *env* reliably detects viral evolution in the PBMCs of untreated patients

**Figure 3:**
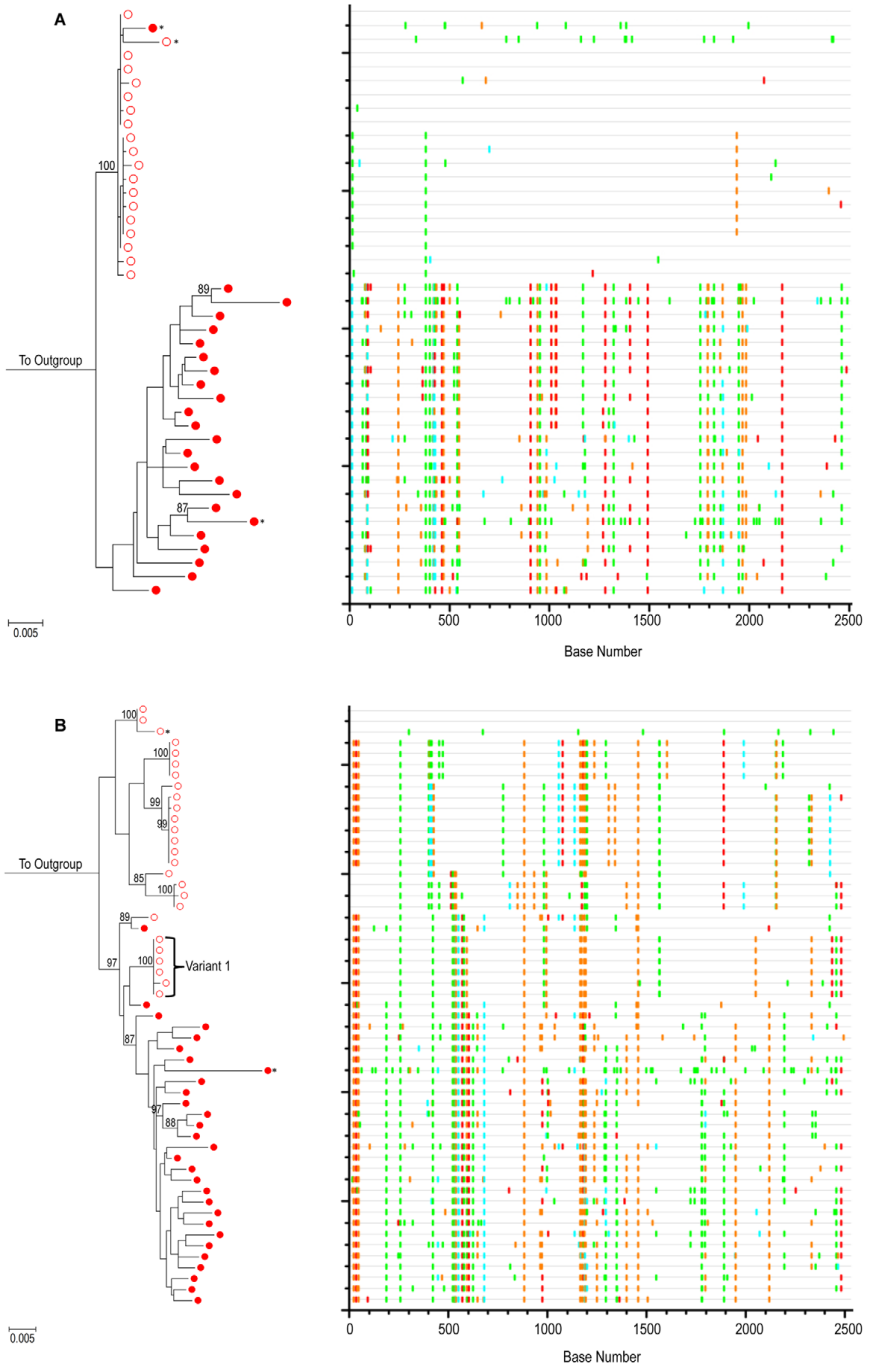
Evolution of HIV-1 *env* in the absence of cART – positive control patients. ML tree of SGA sequences from (A) positive control subject 1 (POS1) and (B) positive control subject 2 (POS2). For both panels, PBMC time point #1 (open red circles) and PBMC time point #2 (closed red circles) are shown. 1000 bootstrap replicates were run and Bootstrap values over 85% are shown. The scale bar represents 0.005 nucleotide substitutions per site. HXB2 was used as an outgroup. Starred sequences represent those determined to be hypermutated. On the right is a Highlighter plot corresponding to the ML tree (outgroup sequence not shown). Individual nucleotide changes from a master sequence (TP1 sequence at the top of the ML tree) are shown. A-green; T-red; G-orange; C-light blue. For participant POS2, phylogenetic analysis reveals acquisition of more than one variant as shown.

To demonstrate our ability to detect measurable viral evolution when expected, we generated HIV-1 *env* SGS from the PBMCs of two individuals who chose not to initiate cART during primary HIV-1 infection. For these two cases, a total of 98 confirmed SGS were obtained (43 for POS1 and 55 for POS2). *Highlighter* plots (http://www.hiv.lanl.gov) were used to visualize individual nucleotide polymorphisms within sequences under consideration [77]. Both phylogenetic trees and *Highlighter* plots for each intra-patient viral variant for POS1 and POS2 are shown (Figure 3). For both individuals, SGA-derived full-length HIV-1 *env* sequences unequivocally revealed that over time, in the absence of cART, there is predictable diversification away from the virus population initiating the infection.

The Tamura-Nei substitution model in MEGA 4.0.2 [78] was used to calculate average pair-wise distances (APD) between intra-patient HIV-1 *env* quasispecies. These calculations allowed for the quantification of genetic diversity within and between PBMC populations at each intra-patient time point. Several sequences were found to have a high statistical likelihood (*p*<0.05) of G→A hypermutations using the Hypermut program available via the Los Alamos website [79]. In contrast to true sequence evolution, hypermutations are believed to be the product of a single replication cycle and as such, they cannot be regarded as evidence of gradual sequence evolution [80], [81]. As a result, these sequences were excluded from quantitative determinations of genetic diversity. For POS1 the average within-patient *env* nucleotide diversity at PBMC time point #1 was 0.13% and increased to 1.23% by the time of the second PBMC sampling 1.9 years later. For POS2, the within-patient *env* nucleotide diversity at PBMC time point #1 was 1.11%. It has been estimated that the maximum within-patient diversity plausibly developing from infection with a single viral variant within 100 days is approximately 0.6% [63]. The within-patient *env* nucleotide diversity of 1.11% within 54 days of infection in POS2 therefore suggests the transmission of more than one viral variant in this case. Examination of the maximum likelihood (ML) phylogenetic tree and *Highlighter* analysis (Figure 3) for this individual was consistent with this hypothesis, as several distinct sequence clusters with ML bootstrap values >85% supported the transmission of more than one viral variant. The high multiplicity of HIV-1 infection in this individual is compatible with previous results by Li et al, in which SGA of HIV-1 *env* revealed 36% of their men who have sex with men (MSM) cohort to have evidence of productive infection with more than one HIV-1 Subtype B virus [82]. At the time of the second PBMC time point 1.8 years later, the within-patient *env* nucleotide diversity in this individual was found to be 1.08%. To quantify the amount of evolution that occurred during the time interval studied, we determined the APD between SGS obtained from ARV naïve time points #1 and #2. For POS1, the experimentally observed HIV-1 *env* nucleotide diversity between the two time points was 1.68%. For POS2, given the evidence of infection with more than one viral variant, we conservatively compared the APD between a monophyletic subset of time point #1 SGS phylogenetically closest to the time point #2 sequences (labeled Variant 1 in Figure 3) and the time point #2 sequences. Using this approach, the HIV-1 *env* nucleotide diversity between the two time points was 1.36%. Given a published rate of evolution of the C2-V5 region of the HIV-1 *env* gene of approximately 1.0% per year [83], the experimentally observed *env* nucleotide diversity between PBMC time points #1 and #2 for each individual are within the expected range for evolving viral quasispecies over a period of 1.8 (POS2) to 1.9 (POS1) years. Altogether, these results demonstrate our ability to detect evolution using SGA when multiple replication cycles have occurred in untreated patients.

### Maximum-likelihood phylogenetic trees of SGA-derived HIV-1 *env* do not reveal viral evolution in the GALT or PBMC of patients during suppressive cART

As detailed above, the three study subjects achieved a significant degree of immune reconstitution in the peripheral blood based on CD4+ T cell count. We hypothesized that if persistent immune activation and CD4+ T cell depletion in the GALT was the result of local ongoing virus replication, evolution of GALT-derived HIV-1 *env* would be most likely to occur in the individual with the highest levels of residual immune activation and lowest level of immune reconstitution despite cART. Therefore, we anticipated that if we were to observe measurable evolution of viral quasispecies during cART, that two scenarios would be apparent: (1) The amount of evolutionary divergence between GALT-derived HIV-1 *env* populations sampled 1–2 years apart would be greater than between longitudinal, contemporaneous PBMC-derived HIV-1 *env* populations and (2) The amount of genetic diversity developing in an individual's GALT-derived HIV-1 *env* quasispecies over time would correlate positively with the level of immune activation and CD4+ T cell depletion measured experimentally. As a result, we would expect the greatest amount of within-patient evolution of HIV-1 viral quasispecies to occur in HIA, followed by IIA, with the lowest levels of evolution in LIA.

**Figure 4:**
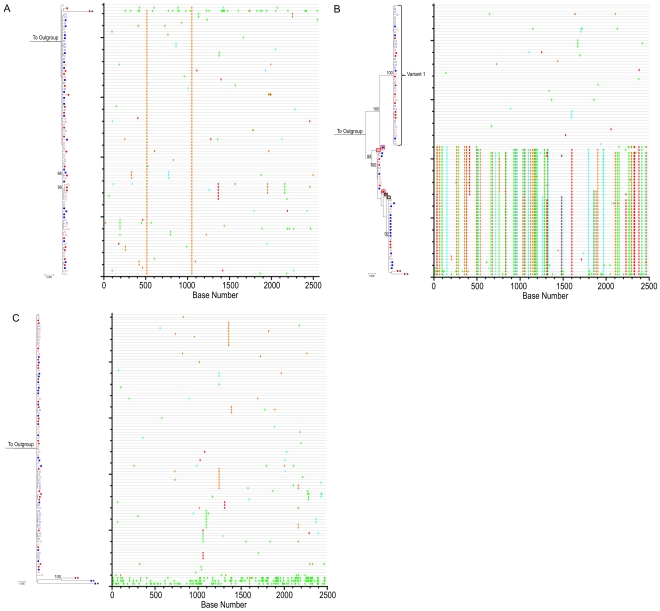
HIV-1 *env* phylogenies do not suggest measurable evolution on suppressive cART. ML tree of SGA sequences from participants (A) HIA (B) IIA and (C) LIA. For all panels, PBMC time point #1 (open red circles), PBMC time point #2 (closed red circles), GALT time point #1 (open blue squares) and GALT time point #2 (closed blue squares) are shown. 1000 bootstrap replicates were run and Bootstrap values over 85% are shown. The scale bar represents 0.005 nucleotide substitutions per site. HXB2 was used as an outgroup. Starred sequences represent those determined to be hypermutated. To the right of each ML tree is the corresponding Highlighter plot (outgroup sequence not shown). Individual nucleotide changes from a master sequence (TP1 sequence at the top of the ML tree) are shown. A-green; T-red; G-orange; C-light blue. For participant IIA, phylogenetic analysis reveals acquisition of more than one variant with *in-vivo* inter-lineage recombinants. Sequences surrounded by boxes represent HIV-1 *env* recombinants (red boxes denote a sequence p value of *p*<0.05 in Recco; black boxes denote a sequence p value of *p*<0.25 in Recco).

Phylogenetic analyses were first used to estimate sequence divergence as described for the positive control cases. Figure 4 shows the ML trees with bootstrap values for HIV-1 *env* sequences derived from pre- and post-treatment PBMC and GALT for participants HIA, IIA and LIA. Corresponding *Highlighter* plots allow for visualization of individual nucleotide polymorphisms within sequences under consideration [77]. Visual inspection of maximum likelihood phylogenetic trees and *Highlighter* plots of full length HIV-1 *env* clearly reveal highly homogeneous viral populations during early HIV-1 infection in both the PBMC and GALT immediately prior to the initiation of cART for individuals HIA and LIA. This is consistent with productive infection in these individuals with a single virus. In each individual, there is a high degree of similarity between SGA-derived HIV-1 *env* sequences from each compartment within the viral variants establishing primary infection. In contrast to what is observed in the absence of cART, the population structure of these ML trees demonstrates no visual evidence of evolutionary diversification away from the virus populations identified during primary infection after 1–2 yrs of suppressive cART in either the PBMC or GALT. Using the same sequence alignments, phylogenetic trees were also generated using the neighbor-joining method [84] implemented in the MEGA program Version 4.0 [78] (data not shown). Bayesian estimation of phylogenies for experimental and control individuals were also generated using MrBayes 3.1.2 [85] yielding similar population structure results (Figure S1).

SGA followed by phylogenetic analysis indicates that individual IIA was productively infected with a minimum of two, and possibly three distinct HIV-1 viral variants. In this individual, a small number of *in-vivo* inter-lineage recombinant viruses (Figure 4B and Table S1) were detected through visual interrogation of the *Highlighter* plot. The Hudson-Kaplan test [86] implemented in the DnaSP software [87] found a minimum of 9 recombination breakpoints among the recombinant sequences. Recombination breakpoints were confirmed and corresponding levels of significance determined using the Recco program [88] (Table S1). The noted recombinants were observed in both the PBMC and GALT and at both time points analyzed. Recombination of HIV-1 *env* quasispecies results in non-clock genetic evolution that can incorrectly bias quantitative estimates of population divergence [89]. Additionally, owing to their relative rarity in the population of HIV-1 *envs* sampled from participant IIA, the recombinants sampled 1–2 years into cART are just as likely to have been present at the initial sampling time point and therefore do not represent evidence of viral evolution during suppressive cART.

### Quantitative estimates of HIV-1 *env* population diversity and divergence do not reveal viral evolution in the GALT or PBMC of experimental patients during suppressive cART

Evidence for the absence of HIV-1 *env* evolution in the PBMC and GALT of the three experimental individuals, regardless of level of residual GALT immune activation or CD4+ T cell depletion is readily found in the phylograms and *Highlighter* plots (Figure 4). In an effort to quantify evolutionary relationships represented in the phylogenetic trees, we determined estimates of population diversity and divergence over sequence pairs within and between groups by measurement of average pairwise distances (APDs). As in the positive control group, sequences with a significant probability of hypermutation were excluded from this analysis.

To detect statistically significant shifts in population structure that might not be evident from APD calculations alone, we used a nonparameteric test for panmixia [90]. This test was derived from a geographic subdivision detection test proposed by Hudson et al. [91]. In an investigation of viral evolutionary divergence in the SIV/pigtail macaque model [92], *p*<10^4^ was used as a significance threshold for panmixia. This stringent *p*- value threshold was chosen to correct for multiple nucleotide comparisons and make panmixia detection robust to potential sampling error by SGS [92]. In this analysis, we have instead chosen to fix the *p* value threshold for panmixia at *p*<0.05, apply a Bonferroni correction to *p*-values obtained from testing of multiple compartments across time points, and require a second, confirmatory test to infer true shifts in population structure in a sample.

The second test used for analysis of population structure was the Slatkin-Maddison (SM) test [93]. Implemented in HyPhy [94]. Given an input tree and predefined groups of sequences in that tree, the SM test uses parsimony to determine the number of migration events, i.e. the number of times a sequence from one group migrated to another group; the observed number of migrations - the degree of intercompartment segregation - is then compared to a null distribution obtained by repeated shuffling (1000 iterations) of the group assignments. This approach applies a parsimony criterion to the evolution of each character on the maximum likelihood gene phylogeny in question, and assesses the degree of variation from the normal distribution of simulated sequences over the tree to assess the degree of intercompartment segregation.

The number of base substitutions per site averaged over all sequence pairs both within and between groups is shown as well as results for tests of panmixia (Tables 2 and 3). As the diversity between viral lineages at transmission represents mutations accumulated in the donor, the determination of viral evolution as a function of changes in genetic diversity is only valid when comparing diversification away from known parental variants. As a result, for participant IIA, only one distinct group of sequences corresponding to one of the multiple infecting viral variants demonstrated in Figure 4B was considered following the removal of both hypermutated and recombinant viral variants: population (Variant 1) with 48 SGS (Figure 4B and Table 3). Given the need to further distinguish between SGA-derived HIV-1 *env* quasispecies obtained from PBMC pre- and post-cART and GALT pre- and post-cART, remaining populations contained too few sequences in each compartment for meaningful quantitative and statistical analyses. As a result, only the largest of the IIA infecting populations (Variant 1) was analyzed (Table 3). Similarly, given evidence of multiple infecting viral variants for POS2 (each with a small number of variants), statistical analysis for POS2 could not be performed (Table 2).

**Table 2:** Nucleotide distance calculations with panmixia and Slatkin-Maddison (SM) probabilities-positive controls.

Participant	Compartment	PBMC TP1		Within Group APD	*p* SM	#SGS
		BG APD	*p* Panmixia			
POS1	PBMC TP1	-	-	0.0013	**<0.001** * ** [<0.001** * **]**	18 [12]
POS1	PBMC TP2	0.0168	**<0.0001** *	0.0123	-	22 [22]
POS2	PBMC TP1	-	-	0.0004	N/A	6
POS2	PBMC TP2	0.0136	N/A	0.0108	-	28

Nucleotide Distance Calculations with panmixia and Slatkin-Maddison (SM) probabilities for positive control (POS1 and POS2) patients. BG = Between Group, APD = Average Pairwise Distance and SM = Slatkin-Maddison Test. #SGS = number of sequences used per compartment in analysis after exclusion of hypermutated and recombinant sequences. For probability of panmixia, *p*-values<0.05 are considered significant. For POS2 analysis for only one infectious variant is shown. #SGS in brackets for POS1 denote number of unique sequences used in Slatkin-Maddison Test. Bracketed Slatkin-Maddison probabilities denotes *p* SM following removal of duplicate sequences.

* = Statistically significant; N/A = Not applicable.

**Table 3:** Nucleotide distance calculations with panmixia and Slatkin-Maddison (SM) probabilities – experimental cases.

Participant	Compartment	PBMC TP1		PBMC TP2		GALT TP1		Within Group APD	*p* SM	#SGS
		BG APD	*p* Panmixia	BG APD	*p* Panmixia	BG APD	*p* Panmixia			
HIA	PBMC TP1	-	-	-	-	-	-	0.0011	0.589	26
HIA	PBMC TP2	0.0013	1.0	-	-	-	-	0.0015	-	19
HIA	GALT TP1	0.0010	1.0	0.0012	0.526	-	-	0.0009	-	22
HIA	GALT TP2	0.0009	1.0	0.0011	1.0	0.0008	**0.027** *	0.0007	-	23
IIA	PBMC TP1	-	-	-	-	-	-	0.0006	0.078	14
IIA	PBMC TP2	0.0006	0.206	-	-	-	-	0.0005	-	10
IIA	GALT TP1	0.0006	0.38*2*	0.0005	0.134	-	-	0.0005	-	20
IIA	GALT TP2	0.0004	N/A	0.0004	N/A	0.0004	N/A	0.0002	-	4
LIA	PBMC TP1	-	-	-	-	-	-	0.0012	**<0.001** * [0.086]	27 [19]
LIA	PBMC TP2	0.0010	0.385	-	-	-	-	0.0008	-	22 [13]
LIA	GALT TP1	0.0013	**0.042** *	0.0012	**0.005** *	-	-	0.0014	-	26 [23]
LIA	GALT TP2	0.0009	0.171	0.0008	0.157	0.0011	**0.003** *	0.0007	-	19 [10]

Nucleotide Distance Calculations with panmixia and Slatkin-Maddison (SM) probabilities for experimental patients (HIA, IIA and LIA) are shown. BG = Between Group, APD = Average Pairwise Distance and SM = Slatkin-Maddison Test. #SGS = number of sequences used per compartment in analysis after exclusion of hypermutated and recombinant sequences. For probability of panmixia, *p*-values<0.05 are considered significant. For IIA, analysis for only one infectious variant is shown. #SGS in brackets for LIA denote number of unique sequences used in Slatkin-Maddison Test. Bracketed Slatkin-Maddison probabilities denotes *p* SM following removal of duplicate sequences.

* = Statistically significant; N/A = Not applicable.

In the event of ongoing viral replication resulting in viral evolution, we would expect to document the all of the following as seen for POS1: (1) Increasing nucleotide diversity within groups over time (increases in within-group APD between time point #1 (TP1) and time point #2 (TP2); (2) Between group (BG) APDs consistent with nucleotide divergence between groups over time and; (3) Significant *p*- values for tests of panmixia reflecting significant population divergence over time. (4) Significant *p*-values for confirmatory Slatkin-Maddison test reflecting true differences in population structure.

Average pairwise distances within PBMC groups during early HIV-1 infection for HIA, IIA (infecting variant #1) and LIA were expectedly low (0.0011, 0.0006 and 0.0012 respectively) (Table 3). At the second time point after 1.1 to 1.3 years of suppressive cART, within group APDs remained low for each individual but were slightly increased for HIA (0.0015) and decreased for both IIA (0.0005) and LIA (0.0008). In each individual, tests of panmixia between PBMC TP1 and PBMC TP2 yielded non-significant *p*-values, indicating a clear mixing of populations and providing assurance that the measured values for between group APDs (BG APDs) were not large enough to represent statistically significant nucleotide divergence between the populations. As a result, we conclude that the small increase in within group APD from PBMC TP2 SGS for HIA is not large enough to represent evidence of viral evolution over time, and the small decrease in within group APD from PBMC SGS for IIA and LIA does not represent evidence of loss of viral diversity over time.

Average pairwise distance determinations within and between GALT groups for HIA reveal a similar story. During early HIV-1 infection, GALT populations in HIA, IIA (Variant 1) and LIA were also homogenous, with low APDs within populations of infecting quasispecies (0.0009 for HIA, 0.0005 for IIA Variant 1 and 0.0014 for LIA). In the case of HIA, the average within-GALT nucleotide distance prior to the initiation of cART was 0.0009. Following 1.3 years of cART, the average within-group nucleotide distance was 0.0007. Sequence compartmentalization between GALT TP1 and TP2 was supported by the test of panmixia (*p* = 0.027). However, confirmatory testing using the Slatkin-Maddison test revealed no significant evidence of population structure in the GALT compartment (*p* = 0.780) or over the entire phylogenetic tree as a whole (*p* = 0.589). This finding, combined with the low APD between GALT TP1 and TP2 (0.0008) and absence of an increase in GALT viral diversity allowed us to conclude that the numerical decrease in the within group APD was not reflective of a significant loss of viral diversity in the GALT of participant HIA at the second time point, and that there was no evidence of HIV-1 *env* evolution in the GALT from the individual we hypothesized most likely to exhibit residual HIV-1 replication in this compartment given their immunologic profile 1.3 years after the initiation of cART (highest degree of GALT immune activation and lowest degree of CD4+ T cell reconstitution).

For IIA, we saw a similar decrease in within group APD between GALT TP1 (0.0005) and GALT TP2, (0.0002) arguing against any evidence of viral evolution in this compartment due to viral replication. However, given the small number of SGS in GALT TP 2 for this variant (<10 SGS), the high potential for sampling error prevented us from performing a statistical test of panmixia between these two groups. As a result, we were unable to determine if the measured decrease in within-group GALT TP2 viral diversity was indicative of a significant loss of diversity over time. Slatkin-Maddison testing for the IIA Variant 1 phylogram (with GALT TP2 sequences removed) was non-significant (*p* = 0.078).

Finally, for LIA, the within-group APDs for GALT TP1 (0.0014) and GALT TP2 (0.0007) SGSs again fails to reveal an increase in HIV-1 *env* diversity over time. However, the panmixia probability for this compartment is significant (*p* = 0.003) and confirmed by Slatkin-Maddison testing (*p*<0.001) of the compartment, and of the complete phylogram (*p*<0.001). Statistical tests of population structure across all compartments and across all time points for this individual reveals significant compartmentalization using both the panmixia and Slatkin-Maddison tests only for those compartments that are compared against GALT TP1. Evaluation of the phylogram and *Highlighter* plots demonstrates a number of identical (monotypic) sequences. Given this observation, and the knowledge that statistical estimates of compartmentalization can be biased by identical sequences [95]–[98], we then collapsed identical sequences into a single sequence within each compartment, at each time point. Repeat Slatkin-Maddison testing on the reconstructed maximum-likelihood phylogram revealed the absence of any significant population structure in the tree (*p* = 0.086). As mentioned earlier, evaluation of pre-treatment plasma and proviral DNA sequences revealed that patient LIA was infected with a dual-tropic HIV-1 variant capable of using both CCR5 and CXCR4 for cellular entry. The extent, if any, to which this may have influenced the expansion of pre-treatment variants in the GALT, where the target cells are overwhelmingly CD4+CCR5+ is unclear.

When compared to their corresponding PBMC population at TP1, low between group APDs and non-significant *p*-values for tests of panmixia reveals the absence of compartmentalization between HIV-1 *env* variants in PBMC and GALT during early infection with HIV-1 for individuals HIA and IIA (Variant 1). Following removal of duplicate sequences in the phylogeny, this is true for LIA as well.

Taken together, in concert with the corresponding maximum-likelihood phylogenies and *Highlighter* plots, these results provide no evidence of significant evolution of HIV-1 *env* in the PBMC or GALT of individuals initiating cART during early HIV-1 infection. Additionally, this data provides no evidence that levels of immune activation in the GALT at time points during which viral suppression has been achieved in the periphery is associated with or predictive of any degree of viral evolution.

## Discussion

In this study we have shown that in a group of HIV-1 infected individuals initiating therapy during early infection, no evidence of substantial viral evolution could be found in HIV-1 *env* variants derived from the peripheral blood mononuclear cells or gut-associated lymphoid tissue after 1–2 years of suppressive cART. We initially hypothesized that if continued HIV-1 replication in the GALT was responsible for persistent immune activation in this compartment, the amount of HIV-1 *env* evolution would correlate positively with experimentally determined levels of GALT immune activation, while correlating negatively with levels of immune reconstitution. In the absence of quantifiable evolution in any of the three carefully selected individuals characterized as having High, Intermediate, or Low levels of Immune activation, we find no evidence to support our initial hypothesis. In an effort to make the search for evolution as rigorous as possible, this research utilized the single genome amplification (SGA) technique to generate HIV-1 variant sequences. SGA has been shown to decrease Taq-induced recombination, template resampling, nucleotide misincorporation and cloning bias when compared to more conventional sequencing methods [55]–[57], [60]–[62]. The use of this method therefore produces a more accurate representation of *in-vivo* HIV-1 quasispecies and this work represents the first application of the SGA method to an evolutionary study of GALT-derived HIV-1 full-length HIV-1 *env* populations.

Controversy in the field regarding the ability of suppressive cART to prevent ongoing cycles of HIV-1 replication remains. In the recent literature, the majority of studies using phylogenetic methods have suggested the absence of HIV-1 evolution in patients undergoing suppressive cART [51]–[53] although dissenting findings exist [45]. Recent literature using alternative experimental methods such as measuring the accumulation of 2-LTR circles in the periphery during intensification of cART-suppressed individuals suggests ongoing replication [41]. These findings however, are also controversial, as other recent intensification studies in patients on suppressive cART have shown no effect on endpoints such as plasma HIV-1 RNA levels or T cell activation in the PBMCs or sigmoid colon in HIV-infected patients with a suboptimal CD4+ T cell response [99] as well as no effect on HIV-1 RNA levels or 2-LTR circles in the plasma [100]. Examining the effect of intensification of suppressive cART with the integrase-inhibitor raltegravir, Yukl *et al.* concluded that in addition to effecting no significant decrease in HIV-1 RNA in plasma, no HIV-1 RNA decreases were noted in the duodenum, colon or rectum [101]. In contrast, the authors did report a decrease in unspliced HIV-1 RNA per 10 CD4+ T cells in the ileum in the majority of patients studied. These data suggest that if the GI tract does have the ability to act as a reservoir for HIV-1 replication, that ability may be compartmentalized to areas outside of the recto-sigmoid colon investigated in our work. One may also consider that although robust phylogenetic studies may not reveal evidence of viral evolution during cART, the ability to identify ongoing HIV-1 replication during suppressive cART may be possible using other experimental methods.

With regards to the more specific question of whether or not the GALT serves as a reservoir for continued viral replication in the presence of cART, our findings are consistent with those of two recent phylogenetic studies examining this unique compartment. Imamichi et al. [102] examined compartmental differences between two sites in the gut (colon and terminal ileum) and peripheral blood in chronically infected HIV+ individuals. Contemporaneous intra-patient clonal sequences spanning the C2-V3 region of the HIV-1 *env* gene were examined from cell-associated DNA and RNA and virion RNA. Phylogenetic analysis revealed no evidence of compartmentalization of HIV-1 between the gut and peripheral blood and in two individuals, neighbor-joining trees revealed no indication of viral evolution during 12 months of suppressive cART. Additionally, Lerner et al. conclude through the bulk sequencing of GALT-derived viral variants rebounding from individuals interrupting cART after initiation of cART during primary HIV-1, that GALT was unlikely to be a major contributor to post-interruption plasma viremia [103].

In the presence of cART, the plasma viral burden is decreased from 4–6 log in our study participants. As a result, the scale of expected evolutionary change is perhaps less than would be expected in the absence of ART. Several studies have looked at HIV-1 *env* replication at this lower limit of detection and their findings are informative. Mens *et al.* looked at evolutionary rates of HIV-1 *pro-rt* and *env* in longitudinal samples from HIV-1 controllers with median plasma HIV-1 RNA levels of 0.3 to 0.8 copies/ml and found evidence of ongoing replication [89] based on increasing viral divergence over time. In addition, while levels of viremia in the HIV-1 non-controllers was significantly higher than that of the HIV-1 controllers studied, the measured rates of evolution of HIV-1 *env* were not very different in the groups. Also, in a recent study by Anderson et al. [104] SGS-derived HIV-1 *envs* from the plasma of two individuals on suppressive cART with low level viremia was found to be identical to HIV-1 *env* sequences recovered from outgrowth assays from pools of resting CD4+ T cells. Finally, in the SIV model, Kearney et al. found that in well-suppressed pathogenic SIV infection, those animals with SIV RNA viral load <20copies/ml showed no evidence of evolution in the SIV *pol* in plasma over a 20 week period [92].

The extent to which the dual tropic nature of the infecting virus in the low immune activator (LIA) patient may have influenced the overall burden of HIV-1 infection in the GALT and subsequent levels of immune activation in that compartment (given that the preferred targets for HIV-1 infection are believed to be mucosal CCR5+ memory CD4+ T cells [20], [27], [28]) is unclear. While removal of duplicate sequences in the LIA phylogram abolished any statistical evidence of compartmentalization between tissues (PBMC vs GALT) and time points (pre- and during- cART), we also must consider the extent to which the elimination of these sequences may have altered the power to detect real differences in the dataset. Known differences in HIV-1 co-receptor expression on CD4+ T cells of the peripheral blood and GI tract, coupled with the dual tropic nature of the infecting viral population in this individual could make compartmentalization at the time of infection between variants in the PBMC and GALT a possibility. Persistence would in our minds more likely reflect a founder effect as opposed to evidence of the generation of viral diversity over time given the absence of evolution on the phylogram, as well as a failure of the sequences to increase in diversity. It is also interesting that for subject IIA, there is an apparent difference in distribution of sequence types among the two or three potential clusters between GALT TP1 and GALT TP2. Based on the amount of dilution necessary to generate single genomes, HIV DNA levels in the GALT of this individual were approximately ∼80× lower in TP2 than in TP1. We hypothesize that this apparent shift in GALT populations may be a reflection of selective clearance of target cells (perhaps a particular subtype of target cell) in the GALT initially harboring a population of viral variants. We do also have to consider, particularly in the face of dramatic declines in HIV-1 DNA in this compartment, that the apparent phylogenetic shift may reflect under-sampling of the compartment.

Limitations of the present study include the generation of sequence data using genomic DNA as the template. This was experimentally necessary; given the need to generate multiple HIV-1 *env* variants from GALT specimens obtained in the setting of plasma HIV-1 RNA levels <50 copies/ml. As a result, one must consider that the sampling of approximately 20 to 25 DNA sequences per tissue per time point from a provirus population may have hindered our ability to adequately detect and sample from the small subset of infected cells containing replication competent virus. Use of viral RNA would more accurately limit the population sampled to those *env* sequences derived from replication competent virus at the time of sampling. However, inclusion of archived variants in the studied population should not have been expected to preclude the discovery of evolved variants if they existed. It is also important to note the small number of subjects studied to date (3 experimental and 2 positive control individuals), as well as the relatively short duration of cART in these studies. Characterization of the study individuals using immune activation phenotypes at only one time point on suppressive cART, as well as the absence of more than one time point following viral suppression for the generation of SGS are further experimental limitations of note. Additional work on the generation of a model of expected levels of evolution in the presence of HAART would be informative. Furthermore, the experiments we report here involve the SGA of full length HIV-1 *env*. This method was chosen because it interrogates a significant fraction (>2.5 kb) of the viral genome that has been shown to evolve at a measurable rate in a number of publications utilizing the SGA technique. We cannot, however, rule out the possibility that we might have been able to document evidence of evolution in other regions of HIV-1 such as *gag*, *pol* or *nef* had they been interrogated. Also, as mucosal biopsy sampling does lead to some degree of bleeding into the tissues, we cannot fully exclude the possibility of some contamination of GALT tissue samples with contemporaneous PBMC. While this may lead to appearance of PBMC derived SGS appearing in the GALT compartment, it does not explain the absence of evolving forms from GALT founder sequences.

Finally, an important consideration in these studies is the unique nature of the study population. In each of the cases, participants were identified during acute/early HIV-1 infection, and in the case of the experimental subjects, initiated cART during this period. Typically, HIV-1 seropositive patients are identified during the chronic phase of infection. It is possible that the findings in our group of individuals cannot be completely generalized to a greater population initiating cART at later time points, with a higher degree of pre-treatment diversity and potentially larger pool of latent CD4+ T cells. However, for this work, the study of individuals initiating therapy during primary infection is preferred, as interpretation of longitudinal nucleotide changes is greatly facilitated by knowledge of the viral quasispecies present close to the time of viral acquisition.

In summary, our data is consistent with the observations made by others suggesting that the success of cART is due to the complete or nearly complete suppression of viral replication achieved with cART [105], [106]. Using the robust technique of SGS of full length HIV-1 *env*, we were unable to identify evolved forms in the PBMC or GALT of individuals with known immunologic evidence of residual GALT immune activation despite clinical evidence of plasma HIV-1 RNA suppression. The absence of evidence of evolved HIV-1 variants during cART described in the present study supports the conclusion that at a minimum, currently available regimens of suppressive cART have the ability to abrogate *de-novo* rounds of HIV-1 replication in the gastrointestinal lymphoid tissue in individuals initiating such therapy during primary infection.

## Materials and Methods

### Ethics statement

This study was approved by the Institutional Review Board of the Rockefeller University (New York, New York, United States). All participants provided written informed consent prior to sample acquisition and all clinical investigation was conducted according to the principles expressed in the Declaration of Helsinki.

### Study subjects and sample acquisition

Study subjects were chosen from the Aaron Diamond AIDS Research Center Acute Infection Program. All study individuals self-identified as men who have sex with men (MSM) and had a clinical history consistent with the acquisition of HIV-1 during sexual contact. Duration of infection at presentation was estimated as 2 weeks prior to the onset of acute retroviral illness.

Serum specimens were tested for viral specific antibodies using the HIV-1/HIV-2 PLUS O enzyme immunoassay (EIA) and Vironostika less sensitive (LS) EIA (the serologic testing algorithm for recent HIV seroconversion [STARHS]). HIV-1 Western Blots were performed by the Public Health Laboratory of the City of New York Department of Health and Mental Hygiene. Plasma samples were tested for HIV-1 RNA and by routine commercial testing battery including the Roche Cobas Amplicor HIV-1 Monitor Test version 1.5 and the Roche COBAS AmpliPrep/ COBAS TaqMan 48 System with AmpliLink Software Version 3.2 as per manufacturer's instructions. These results were used to stage subjects according to the Fiebig classification system for acute and early HIV-1 infection [107].

Contemporaneous peripheral blood and recto-sigmoid colonic mucosal tissue samples were obtained from three cART-naïve, HIV-1 seropositive males identified during acute/early HIV-1 infection. Positive control peripheral blood samples were obtained from two additional cART-naïve, HIV-1 seropositive males identified during acute/early HIV-1 infection. All participants were enrolled with a nonreactive detuned ELISA result or a documented negative HIV-1 test within the 6 months of biopsy.

Endoscopic biopsies were obtained from the colon from macroscopically normal mucosa in all cases via flexible sigmoidoscopy and processed as previously described [30]. Briefly, the biopsies were taken using large-cup endoscopic-biopsy forceps (Microvasive Radial Jaw, Boston Scientific, Boston, Massachusetts, United States) (outside diameter 3.3 mm) and (1) placed immediately in tissue-culture medium (RPMI 1640, Mediatech, Herndon, Virginia, United States); (2) placed into 2-ml pre-labeled cryovials (Nalgene, Rochester, New York, United States) and immediately frozen in liquid nitrogen; or (3) placed in formalin to preserve tissue architecture. Formalin-fixed tissues were washed with phosphate-buffered saline (PBS), transferred to 100% alcohol and processed for immunohistochemistry. Endoscopic biopsies were not obtained for the two positive control participants. Phlebotomy was undertaken immediately prior to endoscopy where applicable.

Immediately after acquisition, mucosal mononuclear cells (MMCs) were enzymatically isolated from mucosal biopsies using a 30-min incubation in collagenase type II (Clostridio-peptidase A, Sigma-Aldrich, St. Louis, Missouri, United States) followed by mechanical separation through a blunt ended 16-gauge needle. The digested cell suspension was strained through a 70-µm disposable plastic strainer. Immediately after isolation, cells were washed with PBS and resuspended in PBS containing antibodies for flow cytometry. Peripheral blood mononuclear cells (PBMCs) were prepared by centrifugation on a Ficoll-Hypaque density gradient (Mediatech).

At protocol defined participant visits, PBMCs were also stored at −70 C in 1 ml of freezing media (10% DMSO 90% FCS) for genomic DNA isolation at a later time.

### Flow cytometry

Cell surface expression of lymphocyte antigens was performed as previously described [30]. Briefly, freshly isolated MMCs were subject to monoclonal antibody staining, followed by flow cytometry using a FACSCalibur (Becton-Dickinson, Palo Alto, California, United States) with analysis using CellQuest software (Becton-Dickinson). Monoclonal antibodies used in this study included: anti-human CD3-fluorescein isothiocyanate (FITC) (clone UCHT1; Becton-Dickinson), anti-human CD3-phycoerythrin (PE) (clone SK-7; Becton-Dickinson), anti-human CD3-peridinin chlorophyll-α protein (PerCP) (clone SK-7; Becton-Dickinson), anti-human CD4-allophycocyanin (clone RPA T4; PharMingen, San Diego, California, United States), anti-human CD8 PE (clone RPA T8; PharMingen), anti-human HLA-DR PerCP (clone L243; BD Biosciences Pharmingen), anti-human CD45RO PE-Cy7 (clone UCHL1, BD Biosciences Pharmingen) and the appropriate isotype controls. During flow cytometry, lymphocytes, initially identified by their forward- and side-scatter characteristics, were subject to phenotypic analysis. Dead cells were excluded from analysis using 7-aminoactinomycin D (Calbiochem, San Diego, California, United States). To determine the percentages of CD4^+^ and CD8^+^ cells in the T cell population, gated lymphocytes were initially examined for the expression of CD3. The CD3^+^ lymphocytes were then analyzed for expression of CD4 and CD8 receptors. To examine for activated memory cells, gated CD8^+^ lymphocytes were examined for the expression of CD45RO and HLA-DR.

### Light microscopy and immunohistochemistry

Endoscopic biopsy tissue sample were fixed in 4% neutral-buffered formalin and embedded in paraffin. Sections (of 5 µm thickness) were cut and stained with hematoxylin-and-eosin and Giemsa stains for light-microscopic evaluation. Immunohistochemistry was performed on paraffin-embedded sections after high-temperature antigen retrieval as previously described [30]. The sections were incubated with 1∶25 dilution of antibody to CD4 (NCL-CD4-IF6, Novocastra Laboratories, Newcastle-upon-Tyne, United Kingdom) or to 1∶100 dilution of antibody to CD8 (C8/144B, DakoCytomaton, Glostrup, Denmark) for 60 min, followed by incubation with a 1∶20 dilution of rabbit anti-mouse secondary antibody (DakoCytomaton code 259) for 20 min. The tertiary antibody (APAAP-Complex Monoclonal Mouse, DakoCytomaton) was applied in 1∶50 dilution. The incubations were carried out at room temperature and were followed by rinsing in Tris-buffered saline (pH 7.4) for 5 min each. The alkaline phosphatase was revealed by New Fuchsin as the chromogen. CD4^+^ or CD8^+^ cells in the LP (effector site) and the organized lymphoid tissue (OLT) (inductive site) were quantified separately. Using a 40× objective, a standard area was set (unit area), and a photomicrograph was taken with a Zeiss AxioImager M1 microscope equipped with AxioCam MRc5 digital camera (Zeiss, Jena, Germany). Fifteen nonoverlapping unit areas were selected for the LP. Using AxioVision (Release 4.5) software (Zeiss), positive cells showing lymphocyte morphology were counted.

### Proviral DNA extraction and Single Genome Amplification

Total genomic DNA from cryopreserved PBMCs from all participants and GALT from experimental participants was extracted by routine methods using the QIAamp DNA Mini Kit (QIAGEN, USA). To minimize the risk of within-patient cross contamination of samples, only one participant sample from one compartment (PBMC or GALT) and time point was processed on any given day.

Following limiting-dilution, single proviral molecules of full-length HIV-1 subtype B *env* gene (>2.5 kb) were amplified by nested PCR using a modification of the SGA method of Salazar-Gonzalez et al. [63], [108]. Briefly, genomic DNA was serially diluted and distributed in replicates of 10 PCR reactions in MicroAmp 96-well plates (Applied Biosystems, Foster City, CA). Poisson distribution dictates that the DNA dilution that yields PCR products in no more than 30% of wells contains one amplifiable DNA template per positive PCR more than 80% of the time [63]. Therefore, genomic DNA was endpoint diluted in 96-well plates such that fewer than 30% of the PCRs yielded an amplification product. Additional PCR amplifications were performed using this dilution in 96-well reaction plates. PCR amplification was carried out in presence of 1× High Fidelity Platinum Taq PCR buffer, 2 mM MgSO4, 0.2 mM each deoxynucleoside triphosphate, 0.2 uM each primer, and 0.025 units/ul of Platinum Taq High Fidelity polymerase in a 20-ul reaction (Invitrogen, Carlsbad, CA). The nested primers for generating full length *env* were as follows: 1^st^ round sense primer *env*5out 5′-TAGAGCCCTGGAAGCATCCAGGAAG-3′, 1^st^ round antisense primer *env*3out 5′- TTGCTACTTGTGATTGCTCCATGT-3′, 2^nd^ round sense primer *env*5in 5′-TTAGGCATCTCCTATGGCAGGAAGAAG-3′ and 2^nd^ round antisense primer *env*3in 5′-GTCTCGAGATACTGCTCCCACCC-3′. PCR parameters were as follows: 94°C for 2 min, followed by 40 cycles of 94°C for 15 s, 58.5°C for 30 s, and 68°C for 4 min followed by a final extension of 68°C for 5 min. The product of the first-round PCR was used as a template in the second-round PCR under same conditions with the following PCR parameters: 94°C for 2 min, followed by 45 cycles of 94°C for 15 s, 61°C for 30 s, and 68°C for 4 min followed by a final extension of 68°C for 15 min. The resulting amplicons were then inspected on a 1% agarose gel (Sigma-Aldrich, St. Louis, MO). All PCR procedures were carried out under clean PCR conditions with appropriate negative controls. A target of 20–30 single-genome sequences were generated for each compartment at each time point. This was followed by direct sequencing of the uncloned amplicons.

### DNA sequencing

HIV-1 *env* gene products were directly sequenced using an automated ABI Prism 3730xl DNA analyzer. (Applied Biosystems, Inc.). Both strands of DNA were sequenced with partially overlapping fragments. All sequencing chromatograms were carefully inspected for sites of ambiguous sequence (double peaks). Sequences for which any chromatogram revealed double peaks were excluded from further analysis, as this was indicative of amplification from more than one template or early Taq polymerase error.

### Sequence alignments

Individual sequence fragments for each amplicon were assembled using the CAP3 DNA sequence assembly program [109]. Multiple alignments of nucleotide sequences were produced using Clustal W [110] with the following parameters: pairwise alignment gap opening penalty 10; gap extension penalty 0.1; multiple alignment gap opening penalty 3; gap extension penalty 1.8. All resulting alignments were inspected and corrected manually using the Alignment Explorer in the MEGA 4.0 software [78] when warranted. Columns with gaps were then removed from the multiple alignments using GapStrip (www.hiv.lanl.gov) [79].

### Determination of coreceptor usage phenotype

In order to characterize co-receptor usage in our patients, V3 loop nucleotide sequences were extracted from multiply aligned full-length HIV-1 *env* for each participant using coordinates 7110–7216 on the HXB2 reference genome via the Gene cutter program on the HIV Los Alamos website www.hiv.lanl.gov. Translated V3 loop sequences were scored using the SINSI position-specific scoring matrix [PSSM] as per Jensen et al. [72].

### Sequence diversity analysis and phylogenetic tree construction

The “distance matrix” calculation in MEGA 4.0.2 [78] was used to determine average pairwise genetic distances within or between compartments. Pairwise distances among HIV-1 env genes were determined using Tamura-Nei substitution model in Mega 4.0.2. Sequences were analyzed for average within compartment-and between compartment diversity. The Findmodel tool on the Los Alamos HIV database site http://www.hiv.lanl.gov/was used to determine the most appropriate nucleotide substitution model for data description. Overall, the phylogenetic model found to best describe the data while allowing for distance matrix calculations to be performed in MEGA 4.0.2 was the Tamura-Nei model [111]. Phylogenetic trees were constructed by the maximum likelihood (ML) method using the Tamura-Nei evolutionary model in the PhyML program [112]. Bootstrap test of phylogeny were performed on 1000 replicates to evaluate the reliability and robustness of each internal branch in the resulting phylogenies. Each set of sequences was then visually inspected using the Highlighter tool available through the Los Alamos HIV website (www.hiv.lanl.gov).

Bayesian Markov Chain Monte Carlo (MCMC) inference of phylogeny was also performed at the nucleotide level using MrBayes version 3.1.2 [85] with the Tamura-Nei nucleotide substitution model of evolution. Neighbor-Joining phylogenies were used as a starting point. Two simultaneous independent runs were performed for each dataset, each with 4 chains of chain length 1×10∧7 sampling every 100 generations. The analyses were run until convergence, as determined by an average standard deviation of split frequencies <0.01. Discarding the first 25% as burn-in, a majority-rule consensus of trees sampled from the posterior distribution was used to derive node support.

### Hypermutation analysis

Enrichment for mutations with APOBEC3G/F signatures was assessed using Hypermut 2.0 (www.hiv.lanl.gov) [79]. For each intra-patient set, the most common form in the first sampled time point was used as the reference sequence. Sequences that yielded a Fischer's exact p-value of 0.05 or lower were considered significantly hypermutated and excluded from analyses of sequence diversity.

### Recombination analyses

Recombinant sequence identification for IIA was performed using Recco [88] and by visual inspection of Highlighter analysis plots. One thousand permutations were run. The method of Hudson and Kaplan [86] as employed in the DnaSP 5.10 software package [87] was used to estimate the minimum number of recombination events required to explain sequence datasets. Sequences with evidence of recombination in Recco were excluded from analyses of sequence diversity.

### Compartmentalization analysis

A nonparameteric test for panmixia was used to calculate shifts in population structure [90]. The online version of this test was applied from the site at http://wwwabi.snv.jussieu.fr/~achaz/hudsontest.html. The online portal allows for the input of intra-patient SGA derived HIV-1 *env* multiple alignments from the compartments (PBMC and GALT) and time points under consideration. This test was derived from a geographic subdivision detection test proposed by Hudson et al. [91] and compares an estimate of the degree of genetic differentiation in subpopulations of SGS chosen for comparison. In the absence of genetic differentiation between subpopulations, random reassignment of SGSs to different groups would be expected to recapitulate a new, imaginary population with population structures with the same distribution as the experimentally observed subpopulation. Ten thousand (10,000) re-labelings/permutations were used to generate a *p*-value for the probability that the randomized SGS-derived population structures between compartments are statistically equivalent.

The Slatkin-Maddison test [93] as implemented in the HyPhy software package [94], was also used to detect population structure amongst HIV-1 *env* sequences within individual ML phylograms as indicated. The significance of group separation was determined using the permutation test (1000 permutations).

### Nucleotide sequence accession numbers

All non-hypermutated HIV-1 *env* sequences discussed in this manuscript have been deposited in GenBank (accession numbers JQ250832-JQ251198).

## Supporting Information

Figure S1 **Bayesian inference (BI) phylogenetic trees from all individuals.** BI trees of SGA sequences from positive control (A) POS1 (B) POS2 and experimental patients (C) HIA (D) IIA (E) LIA. For all panels, PBMC time point #1 (open red circles) and PBMC time point #2 (closed red circles) are shown. For experimental individuals, GALT time point #1 (open blue squares) and GALT time point #2 (closed blue squares) are also shown. Posterior probabilities over 85% are indicated. The scale bar represents 0.005 nucleotide substitutions per site. HXB2 was used as an outgroup. Starred sequences represent those determined to be hypermutated. Sequences surrounded by boxes represent HIV-1 *env* recombinants (red boxes denote a sequence p value of *p*<0.05 in Recco; black boxes denote a sequence p value of *p*<0.25 in Recco).
(TIF)

Table S1 **Patient IIA – sequence recombination breakpoints and levels of significance.** Recombinant sequences identified by manual interrogation of *Highlighter plots* and Recco analysis for patient IIA are listed with recombination breakpoints and corresponding sequence *p*-values. Sequence *p*-values were derived from 1000 permutations. The method of Hudson and Kaplan as employed in the DnaSP 5.10 software package was used to estimate the minimum number of recombination events required to explain sequence datasets.
(DOCX)

## References

[1] Mellors JW, Rinaldo CR, Gupta P, White RM, Todd JA (1996). Prognosis in HIV-1 infection predicted by the quantity of virus in plasma.. Science.

[2] Daar ES (1998). Virology and immunology of acute HIV type 1 infection.. AIDS Res Hum Retroviruses.

[3] Rosenberg ES, Altfeld M, Poon SH, Phillips MN, Wilkes BM (2000). Immune control of HIV-1 after early treatment of acute infection.. Nature.

[4] Mohri H, Perelson AS, Tung K, Ribeiro RM, Ramratnam B (2001). Increased turnover of T lymphocytes in HIV-1 infection and its reduction by antiretroviral therapy.. J Exp Med.

[5] Chun TW, Carruth L, Finzi D, Shen X, DiGiuseppe JA (1997). Quantification of latent tissue reservoirs and total body viral load in HIV-1 infection.. Nature.

[6] CDC (2006). Epidemiology of HIV/AIDS–United States, 1981–2005.. MMWR Morb Mortal Wkly Rep.

[7] Palella FJ, Delaney KM, Moorman AC, Loveless MO, Fuhrer J (1998). Declining morbidity and mortality among patients with advanced human immunodeficiency virus infection. HIV Outpatient Study Investigators.. N Engl J Med.

[8] Zhang ZQ, Notermans DW, Sedgewick G, Cavert W, Wietgrefe S (1998). Kinetics of CD4+ T cell repopulation of lymphoid tissues after treatment of HIV-1 infection.. Proc Natl Acad Sci U S A.

[9] Autran B, Carcelain G, Li TS, Blanc C, Mathez D (1997). Positive effects of combined antiretroviral therapy on CD4+ T cell homeostasis and function in advanced HIV disease.. Science.

[10] Pakker NG, Notermans DW, de Boer RJ, Roos MT, de Wolf F (1998). Biphasic kinetics of peripheral blood T cells after triple combination therapy in HIV-1 infection: a composite of redistribution and proliferation.. Nat Med.

[11] Wong JK, Gunthard HF, Havlir DV, Zhang ZQ, Haase AT (1997). Reduction of HIV-1 in blood and lymph nodes following potent antiretroviral therapy and the virologic correlates of treatment failure.. Proc Natl Acad Sci U S A.

[12] Finzi D, Hermankova M, Pierson T, Carruth LM, Buck C (1997). Identification of a reservoir for HIV-1 in patients on highly active antiretroviral therapy.. Science.

[13] Wong JK, Hezareh M, Gunthard HF, Havlir DV, Ignacio CC (1997). Recovery of replication-competent HIV despite prolonged suppression of plasma viremia.. Science.

[14] Volberding PA, Deeks SG (1998). Antiretroviral therapy for HIV infection: promises and problems.. JAMA.

[15] Markowitz M, Jin X, Hurley A, Simon V, Ramratnam B (2002). Discontinuation of antiretroviral therapy commenced early during the course of human immunodeficiency virus type 1 infection, with or without adjunctive vaccination.. J Infect Dis.

[16] Finzi D, Blankson J, Siliciano JD, Margolick JB, Chadwick K (1999). Latent infection of CD4+ T cells provides a mechanism for lifelong persistence of HIV-1, even in patients on effective combination therapy.. Nat Med.

[17] Chun TW, Engel D, Mizell SB, Ehler LA, Fauci AS (1998). Induction of HIV-1 replication in latently infected CD4+ T cells using a combination of cytokines.. J Exp Med.

[18] Mowat AM (2003). Anatomical basis of tolerance and immunity to intestinal antigens.. Nat Rev Immunol.

[19] Kim SK, Reed DS, Heath WR, Carbone F, Lefrancois L (1997). Activation and migration of CD8 T cells in the intestinal mucosa.. J Immunol.

[20] Schieferdecker HL, Ullrich R, Hirseland H, Zeitz M (1992). T cell differentiation antigens on lymphocytes in the human intestinal lamina propria.. J Immunol.

[21] Veazey RS, DeMaria M, Chalifoux LV, Shvetz DE, Pauley DR (1998). Gastrointestinal tract as a major site of CD4+ T cell depletion and viral replication in SIV infection.. Science.

[22] Smit-McBride Z, Mattapallil JJ, McChesney M, Ferrick D, Dandekar S (1998). Gastrointestinal T lymphocytes retain high potential for cytokine responses but have severe CD4(+) T-cell depletion at all stages of simian immunodeficiency virus infection compared to peripheral lymphocytes.. J Virol.

[23] Harouse JM, Gettie A, Tan RC, Blanchard J, Cheng-Mayer C (1999). Distinct pathogenic sequela in rhesus macaques infected with CCR5 or CXCR4 utilizing SHIVs.. Science.

[24] Mattapallil JJ, Douek DC, Hill B, Nishimura Y, Martin M (2005). Massive infection and loss of memory CD4+ T cells in multiple tissues during acute SIV infection.. Nature.

[25] Veazey RS, Mansfield KG, Tham IC, Carville AC, Shvetz DE (2000). Dynamics of CCR5 expression by CD4(+) T cells in lymphoid tissues during simian immunodeficiency virus infection.. J Virol.

[26] Nabel G, Baltimore D (1987). An inducible transcription factor activates expression of human immunodeficiency virus in T cells.. Nature.

[27] Poles MA, Elliott J, Taing P, Anton PA, Chen IS (2001). A preponderance of CCR5(+) CXCR4(+) mononuclear cells enhances gastrointestinal mucosal susceptibility to human immunodeficiency virus type 1 infection.. J Virol.

[28] Anton PA, Elliott J, Poles MA, McGowan IM, Matud J (2000). Enhanced levels of functional HIV-1 co-receptors on human mucosal T cells demonstrated using intestinal biopsy tissue.. AIDS.

[29] Brenchley JM, Schacker TW, Ruff LE, Price DA, Taylor JH (2004). CD4+ T cell depletion during all stages of HIV disease occurs predominantly in the gastrointestinal tract.. J Exp Med.

[30] Mehandru S, Poles MA, Tenner-Racz K, Horowitz A, Hurley A (2004). Primary HIV-1 infection is associated with preferential depletion of CD4+ T lymphocytes from effector sites in the gastrointestinal tract.. J Exp Med.

[31] Guadalupe M, Reay E, Sankaran S, Prindiville T, Flamm J (2003). Severe CD4+ T-cell depletion in gut lymphoid tissue during primary human immunodeficiency virus type 1 infection and substantial delay in restoration following highly active antiretroviral therapy.. J Virol.

[32] Mehandru S, Poles MA, Tenner-Racz K, Jean-Pierre P, Manuelli V (2006). Lack of mucosal immune reconstitution during prolonged treatment of acute and early HIV-1 infection.. PLoS Med.

[33] Mehandru S, Poles MA, Tenner-Racz K, Manuelli V, Jean-Pierre P (2007). Mechanisms of gastrointestinal CD4+ T-cell depletion during acute and early human immunodeficiency virus type 1 infection.. J Virol.

[34] Chun TW, Engel D, Berrey MM, Shea T, Corey L (1998). Early establishment of a pool of latently infected, resting CD4(+) T cells during primary HIV-1 infection.. Proc Natl Acad Sci U S A.

[35] Palmer S, Wiegand AP, Maldarelli F, Bazmi H, Mican JM (2003). New real-time reverse transcriptase-initiated PCR assay with single-copy sensitivity for human immunodeficiency virus type 1 RNA in plasma.. J Clin Microbiol.

[36] Ramratnam B, Ribeiro R, He T, Chung C, Simon V (2004). Intensification of antiretroviral therapy accelerates the decay of the HIV-1 latent reservoir and decreases, but does not eliminate, ongoing virus replication.. J Acquir Immune Defic Syndr.

[37] Lewin SR, Vesanen M, Kostrikis L, Hurley A, Duran M (1999). Use of real-time PCR and molecular beacons to detect virus replication in human immunodeficiency virus type 1-infected individuals on prolonged effective antiretroviral therapy.. J Virol.

[38] Chun TW, Nickle DC, Justement JS, Large D, Semerjian A (2005). HIV-infected individuals receiving effective antiviral therapy for extended periods of time continually replenish their viral reservoir.. J Clin Invest.

[39] Sharkey ME, Teo I, Greenough T, Sharova N, Luzuriaga K (2000). Persistence of episomal HIV-1 infection intermediates in patients on highly active anti-retroviral therapy.. Nat Med.

[40] Zhang L, Ramratnam B, Tenner-Racz K, He Y, Vesanen M (1999). Quantifying residual HIV-1 replication in patients receiving combination antiretroviral therapy.. N Engl J Med.

[41] Buzon MJ, Massanella M, Llibre JM, Esteve A, Dahl V (2010). HIV-1 replication and immune dynamics are affected by raltegravir intensification of HAART-suppressed subjects.. Nat Med.

[42] Benito JM, Lopez M, Lozano S, Martinez P, Gonzalez-Lahoz J (2004). CD38 expression on CD8 T lymphocytes as a marker of residual virus replication in chronically HIV-infected patients receiving antiretroviral therapy.. AIDS Res Hum Retroviruses.

[43] Gunthard HF, Wong JK, Ignacio CC, Guatelli JC, Riggs NL (1998). Human immunodeficiency virus replication and genotypic resistance in blood and lymph nodes after a year of potent antiretroviral therapy.. J Virol.

[44] Martinez MA, Cabana M, Ibanez A, Clotet B, Arno A (1999). Human immunodeficiency virus type 1 genetic evolution in patients with prolonged suppression of plasma viremia.. Virology.

[45] Shiu C, Cunningham CK, Greenough T, Muresan P, Sanchez-Merino V (2009). Identification of ongoing human immunodeficiency virus type 1 (HIV-1) replication in residual viremia during recombinant HIV-1 poxvirus immunizations in patients with clinically undetectable viral loads on durable suppressive highly active antiretroviral therapy.. J Virol.

[46] Gunthard HF, Frost SD, Leigh-Brown AJ, Ignacio CC, Kee K (1999). Evolution of envelope sequences of human immunodeficiency virus type 1 in cellular reservoirs in the setting of potent antiviral therapy.. J Virol.

[47] Bailey JR, Sedaghat AR, Kieffer T, Brennan T, Lee PK (2006). Residual human immunodeficiency virus type 1 viremia in some patients on antiretroviral therapy is dominated by a small number of invariant clones rarely found in circulating CD4+ T cells.. J Virol.

[48] Dinoso JB, Kim SY, Wiegand AM, Palmer SE, Gange SJ (2009). Treatment intensification does not reduce residual HIV-1 viremia in patients on highly active antiretroviral therapy.. Proc Natl Acad Sci U S A.

[49] Kieffer TL, Finucane MM, Nettles RE, Quinn TC, Broman KW (2004). Genotypic analysis of HIV-1 drug resistance at the limit of detection: virus production without evolution in treated adults with undetectable HIV loads.. J Infect Dis.

[50] Shen L, Siliciano RF (2008). Viral reservoirs, residual viremia, and the potential of highly active antiretroviral therapy to eradicate HIV infection.. J Allergy Clin Immunol.

[51] Sedaghat AR, Siliciano JD, Brennan TP, Wilke CO, Siliciano RF (2007). Limits on replenishment of the resting CD4+ T cell reservoir for HIV in patients on HAART.. PLoS Pathog.

[52] Sedaghat AR, Siliciano RF, Wilke CO (2008). Low-level HIV-1 replication and the dynamics of the resting CD4+ T cell reservoir for HIV-1 in the setting of HAART.. BMC Infect Dis.

[53] Joos B, Fischer M, Kuster H, Pillai SK, Wong JK (2008). HIV rebounds from latently infected cells, rather than from continuing low-level replication.. Proc Natl Acad Sci U S A.

[54] Sigal A, Kim JT, Balazs AB, Dekel E, Mayo A (2011). Cell-to-cell spread of HIV permits ongoing replication despite antiretroviral therapy.. Nature.

[55] Palmer S, Kearney M, Maldarelli F, Halvas EK, Bixby CJ (2005). Multiple, linked human immunodeficiency virus type 1 drug resistance mutations in treatment-experienced patients are missed by standard genotype analysis.. J Clin Microbiol.

[56] Shriner D, Rodrigo AG, Nickle DC, Mullins JI (2004). Pervasive genomic recombination of HIV-1 in vivo.. Genetics.

[57] Simmonds P, Balfe P, Ludlam CA, Bishop JO, Brown AJ (1990). Analysis of sequence diversity in hypervariable regions of the external glycoprotein of human immunodeficiency virus type 1.. J Virol.

[58] Simmonds P, Balfe P, Peutherer JF, Ludlam CA, Bishop JO (1990). Human immunodeficiency virus-infected individuals contain provirus in small numbers of peripheral mononuclear cells and at low copy numbers.. J Virol.

[59] Butler DM, Pacold ME, Jordan PS, Richman DD, Smith DM (2009). The efficiency of single genome amplification and sequencing is improved by quantitation and use of a bioinformatics tool.. J Virol Methods.

[60] Fang G, Zhu G, Burger H, Keithly JS, Weiser B (1998). Minimizing DNA recombination during long RT-PCR.. J Virol Methods.

[61] Meyerhans A, Vartanian JP, Wain-Hobson S (1990). DNA recombination during PCR.. Nucleic Acids Res.

[62] Yang YL, Wang G, Dorman K, Kaplan AH (1996). Long polymerase chain reaction amplification of heterogeneous HIV type 1 templates produces recombination at a relatively high frequency.. AIDS Res Hum Retroviruses.

[63] Salazar-Gonzalez JF, Bailes E, Pham KT, Salazar MG, Guffey MB (2008). Deciphering human immunodeficiency virus type 1 transmission and early envelope diversification by single-genome amplification and sequencing.. J Virol.

[64] Hazenberg MD, Otto SA, van Benthem BH, Roos MT, Coutinho RA (2003). Persistent immune activation in HIV-1 infection is associated with progression to AIDS.. AIDS.

[65] Salazar-Gonzalez J, Pham K, Keele B, McPherson D (2007). Standard Operating Procedure for: Single Genome Amplification of HIV-1 Envelope [SOP#: CHAVI-MBSC-1].. University of Alabama at Birmingham.

[66] Learn GH, Korber BT, Foley B, Hahn BH, Wolinsky SM (1996). Maintaining the integrity of human immunodeficiency virus sequence databases.. J Virol.

[67] Alcantara LC, Cassol S, Libin P, Deforche K, Pybus OG (2009). A standardized framework for accurate, high-throughput genotyping of recombinant and non-recombinant viral sequences.. Nucleic Acids Res.

[68] de Oliveira T, Deforche K, Cassol S, Salminen M, Paraskevis D (2005). An automated genotyping system for analysis of HIV-1 and other microbial sequences.. Bioinformatics.

[69] Choe H, Farzan M, Sun Y, Sullivan N, Rollins B (1996). The beta-chemokine receptors CCR3 and CCR5 facilitate infection by primary HIV-1 isolates.. Cell.

[70] Connor RI, Sheridan KE, Ceradini D, Choe S, Landau NR (1997). Change in coreceptor use correlates with disease progression in HIV-1–infected individuals.. J Exp Med.

[71] Brumme ZL, Dong WW, Yip B, Wynhoven B, Hoffman NG (2004). Clinical and immunological impact of HIV envelope V3 sequence variation after starting initial triple antiretroviral therapy.. AIDS.

[72] Jensen MA, Li FS, van't Wout AB, Nickle DC, Shriner D (2003). Improved coreceptor usage prediction and genotypic monitoring of R5-to-X4 transition by motif analysis of human immunodeficiency virus type 1 env V3 loop sequences.. J Virol.

[73] Richman DD, Bozzette SA (1994). The impact of the syncytium-inducing phenotype of human immunodeficiency virus on disease progression.. J Infect Dis.

[74] Moore JP, Kitchen SG, Pugach P, Zack JA (2004). The CCR5 and CXCR4 coreceptors–central to understanding the transmission and pathogenesis of human immunodeficiency virus type 1 infection.. AIDS Res Hum Retroviruses.

[75] Margolis L, Shattock R (2006). Selective transmission of CCR5-utilizing HIV-1: the ‘gatekeeper’ problem resolved?. Nat Rev Microbiol.

[76] Whitcomb JM, Huang W, Fransen S, Limoli K, Toma J (2007). Development and characterization of a novel single-cycle recombinant-virus assay to determine human immunodeficiency virus type 1 coreceptor tropism.. Antimicrob Agents Chemother.

[77] Keele BF, Giorgi EE, Salazar-Gonzalez JF, Decker JM, Pham KT (2008). Identification and characterization of transmitted and early founder virus envelopes in primary HIV-1 infection.. Proc Natl Acad Sci U S A.

[78] Tamura K, Dudley J, Nei M, Kumar S (2007). MEGA4: Molecular Evolutionary Genetics Analysis (MEGA) software version 4.0.. Mol Biol Evol.

[79] Rose PP, Korber BT (2000). Detecting hypermutations in viral sequences with an emphasis on G→A hypermutation.. Bioinformatics.

[80] Vartanian JP, Meyerhans A, Sala M, Wain-Hobson S (1994). G→A hypermutation of the human immunodeficiency virus type 1 genome: evidence for dCTP pool imbalance during reverse transcription.. Proc Natl Acad Sci U S A.

[81] Martinez MA, Vartanian JP, Wain-Hobson S (1994). Hypermutagenesis of RNA using human immunodeficiency virus type 1 reverse transcriptase and biased dNTP concentrations.. Proc Natl Acad Sci U S A.

[82] Li H, Bar KJ, Wang S, Decker JM, Chen Y (2010). High Multiplicity Infection by HIV-1 in Men Who Have Sex with Men.. PLoS Pathog.

[83] Shankarappa R, Margolick JB, Gange SJ, Rodrigo AG, Upchurch D (1999). Consistent viral evolutionary changes associated with the progression of human immunodeficiency virus type 1 infection.. J Virol.

[84] Saitou N, Nei M (1987). The neighbor-joining method: a new method for reconstructing phylogenetic trees.. Mol Biol Evol.

[85] Ronquist F, Huelsenbeck JP (2003). MrBayes 3: Bayesian phylogenetic inference under mixed models.. Bioinformatics.

[86] Hudson RR, Kaplan NL (1985). Statistical properties of the number of recombination events in the history of a sample of DNA sequences.. Genetics.

[87] Rozas J, Sanchez-DelBarrio JC, Messeguer X, Rozas R (2003). DnaSP, DNA polymorphism analyses by the coalescent and other methods.. Bioinformatics.

[88] Maydt J, Lengauer T (2006). Recco: recombination analysis using cost optimization.. Bioinformatics.

[89] Mens H, Kearney M, Wiegand A, Shao W, Schonning K (2010). HIV-1 continues to replicate and evolve in patients with natural control of HIV infection.. J Virol.

[90] Achaz G, Palmer S, Kearney M, Maldarelli F, Mellors JW (2004). A robust measure of HIV-1 population turnover within chronically infected individuals.. Mol Biol Evol.

[91] Hudson RR, Boos DD, Kaplan NL (1992). A statistical test for detecting geographic subdivision.. Mol Biol Evol.

[92] Kearney M, Spindler J, Shao W, Maldarelli F, Palmer S (2011). Genetic diversity of simian immunodeficiency virus encoding HIV-1 reverse transcriptase persists in macaques despite antiretroviral therapy.. J Virol.

[93] Slatkin M, Maddison WP (1989). A cladistic measure of gene flow inferred from the phylogenies of alleles.. Genetics.

[94] Pond SL, Frost SD, Muse SV (2005). HyPhy: hypothesis testing using phylogenies.. Bioinformatics.

[95] Gantt S, Carlsson J, Heath L, Bull ME, Shetty AK (2010). Genetic analyses of HIV-1 env sequences demonstrate limited compartmentalization in breast milk and suggest viral replication within the breast that increases with mastitis.. J Virol.

[96] Heath L, Fox A, McClure J, Diem K, van't Wout AB (2009). Evidence for limited genetic compartmentalization of HIV-1 between lung and blood.. PLoS One.

[97] Bull M, Learn G, Genowati I, McKernan J, Hitti J (2009). Compartmentalization of HIV-1 within the female genital tract is due to monotypic and low-diversity variants not distinct viral populations.. PLoS One.

[98] Bull ME, Learn GH, McElhone S, Hitti J, Lockhart D (2009). Monotypic human immunodeficiency virus type 1 genotypes across the uterine cervix and in blood suggest proliferation of cells with provirus.. J Virol.

[99] Hatano H, Hayes TL, Dahl V, Sinclair E, Lee TH (2011). A randomized, controlled trial of raltegravir intensification in antiretroviral-treated, HIV-infected patients with a suboptimal CD4+ T cell response.. J Infect Dis.

[100] Gandhi RT, Zheng L, Bosch RJ, Chan ES, Margolis DM (2010). The effect of raltegravir intensification on low-level residual viremia in HIV-infected patients on antiretroviral therapy: a randomized controlled trial.. PLoS Med.

[101] Yukl SA, Shergill AK, McQuaid K, Gianella S, Lampiris H (2010). Effect of raltegravir-containing intensification on HIV burden and T-cell activation in multiple gut sites of HIV-positive adults on suppressive antiretroviral therapy.. AIDS.

[102] Imamichi H, Degray G, Dewar RL, Mannon P, Yao M (2011). Lack of compartmentalization of HIV-1 quasispecies between the gut and peripheral blood compartments.. J Infect Dis.

[103] Lerner P, Guadalupe M, Donovan R, Hung J, Flamm J (2011). Gut mucosal viral reservoir in HIV infected patients is not the major source of rebound plasma viremia following HAART interruption.. J Virol.

[104] Anderson JA, Archin NM, Ince W, Parker D, Wiegand A (2011). Clonal Sequences Recovered From Residual HIV-1 Viremia In Patients On Intensified Antiretroviral Therapy Are Identical To Replicating Viral RNAs Recovered From Circulating Resting CD4+ T Cells.. J Virol.

[105] McMahon D, Jones J, Wiegand A, Gange SJ, Kearney M (2010). Short-course raltegravir intensification does not reduce persistent low-level viremia in patients with HIV-1 suppression during receipt of combination antiretroviral therapy.. Clin Infect Dis.

[106] Siliciano RF (2005). Scientific rationale for antiretroviral therapy in 2005: viral reservoirs and resistance evolution.. Top HIV Med.

[107] Fiebig EW, Wright DJ, Rawal BD, Garrett PE, Schumacher RT (2003). Dynamics of HIV viremia and antibody seroconversion in plasma donors: implications for diagnosis and staging of primary HIV infection.. AIDS.

[108] Salazar-Gonzalez JF, Pham KT, Keele BF, McPherson D (2007). Standard Operating Procedure for: Single Genome Amplification of HIV-1 Envelope [SOP#: CHAVI-MBSC-1].. University of Alabama at Birmingham.

[109] Huang X, Madan A (1999). CAP3: A DNA sequence assembly program.. Genome Res.

[110] Larkin MA, Blackshields G, Brown NP, Chenna R, McGettigan PA (2007). Clustal W and Clustal X version 2.0.. Bioinformatics.

[111] Tamura K, Nei M (1993). Estimation of the number of nucleotide substitutions in the control region of mitochondrial DNA in humans and chimpanzees.. Mol Biol Evol.

[112] Guindon S, Gascuel O (2003). A simple, fast, and accurate algorithm to estimate large phylogenies by maximum likelihood.. Syst Biol.

