# Supplementary figures and images for: Absence of HIV-1 Evolution in the Gut-Associated Lymphoid Tissue from Patients on Combination Antiviral Therapy Initiated during Primary Infection
Source: PLoS Pathog. 2012 Feb 2;8(2):e1002506. doi: 10.1371/journal.ppat.1002506 (PMC3271083; doi:10.1371/journal.ppat.1002506)

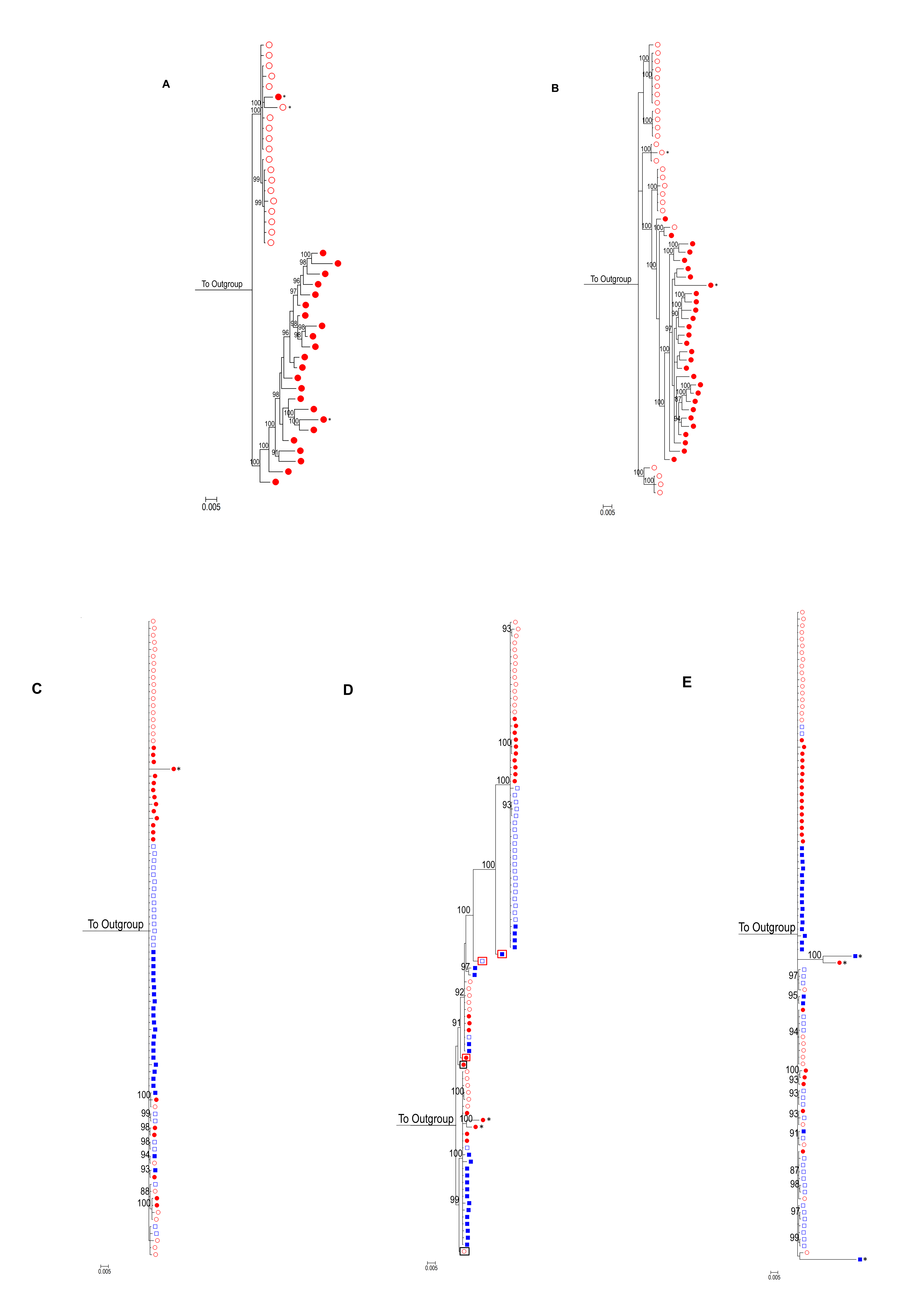

Supplement: Figure S1 — Bayesian inference (BI) phylogenetic trees from all individuals. BI trees of SGA sequences from positive control (A) POS1 (B) POS2 and experimental patients (C) HIA (D) IIA (E) LIA. For all panels, PBMC time point #1 (open red circles) and PBMC time point #2 (closed red circles) are shown. For experimental individuals, GALT time point #1 (open blue squares) and GALT time point #2 (closed blue squares) are also shown. Posterior probabilities over 85% are indicated. The scale bar represents 0.005 nucleotide substitutions per site. HXB2 was used as an outgroup. Starred sequences represent those determined to be hypermutated. Sequences surrounded by boxes represent HIV-1 env recombinants (red boxes denote a sequence p value of p<0.05 in Recco; black boxes denote a sequence p value of p<0.25 in Recco). (TIF) [file ppat.1002506.s001.tif]
