# Supplementary material for: Absence of HIV-1 Evolution in the Gut-Associated Lymphoid Tissue from Patients on Combination Antiviral Therapy Initiated during Primary Infection
Source: PLoS Pathog. 2012 Feb 2;8(2):e1002506. doi: 10.1371/journal.ppat.1002506 (PMC3271083; doi:10.1371/journal.ppat.1002506)
Supplement: Table S1 — Patient IIA – sequence recombination breakpoints and levels of significance. Recombinant sequences identified by manual interrogation of Highlighter plots and Recco analysis for patient IIA are listed with recombination breakpoints and corresponding sequence p-values. Sequence p-values were derived from 1000 permutations. The method of Hudson and Kaplan as employed in the DnaSP 5.10 software package was used to estimate the minimum number of recombination events required to explain sequence datasets. (DOCX) [file ppat.1002506.s002.docx]

**SUPPLEMENTAL TABLE**

**Table S1**. Patient IIA – Sequence Recombination Breakpoints and Levels of Significance

| Sequence | Sequence *p* value | Start | End |
| --- | --- | --- | --- |
| A2G418E9 | <0.001 | 507 | 516 |
| A2G418E9 | - | 2144 | 2162 |
| A1G4118D4 | <0.001 | 551 | 676 |
| A1G4118D4 | - | 849 | 941 |
| A1G4118D4 | - | 1983 | 2119 |
| A2P2288C2 | 0.002 | 1504 | 1869 |
| A1P268E4 | 0.088 | 1272 | 1321 |
| A2P2288F4 | 0.209 | 40 | 416 |
| A2P2288F4 | - | 1504 | 1869 |
